# α,β-Unsaturated Diazoketones as Building Blocks to Piperidine Alkaloids: Total Synthesis of (−)-Cassine

**DOI:** 10.1021/acs.joc.5c00729

**Published:** 2025-06-10

**Authors:** João Pedro de F. Lima, Rafael D. C. Gallo, Antonio C. B. Burtoloso

**Affiliations:** † Institute of Chemistry of São Carlos, University of São Paulo, São Carlos 13560-970, Brazil; ‡ Institute of Chemistry, University of Campinas, Campinas 13083-862, Brazil

## Abstract

The total synthesis of (−)-cassine, a piperidine alkaloid, was accomplished in 10 steps, starting from *N*-Cbz-*O*-TBDPS- *l* -serinal. Key transformations include the preparation of a novel α,β-unsaturated α′-methyl diazoketone, followed by its cyclization into a highly functionalized dihydropyridine-3-one via a *cis*-selective intramolecular N–H insertion reaction. This strategy was further extended to other α′-alkylated α,β-unsaturated diazoketones, enabling the synthesis of new dihydropyridine-3-ones with potential applications in the total synthesis of cassine derivatives.

## Graphical abstract

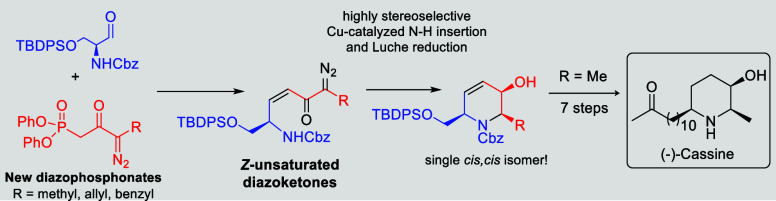

Among the various classes of secondary metabolites, alkaloids^1^ stand out for their vast molecular diversity. (−)-Cassine, a piperidine alkaloid, was first isolated from plants of the Cassia and Senna genera. Discovered in 1964 by R. J. Higuet,^2^ and with its absolute stereochemistry determined by W. Y. Rice two years later,^3^ (−)-cassine has been widely studied for its biological activities, including anti-inflammatory, antibacterial, and anticancer properties.^4^ As a result, (−)-cassine and other 2,3,6-trisubstituted piperidine alkaloids (Figure 1) have become important synthetic targets in organic chemistry.

**Figure 1:**
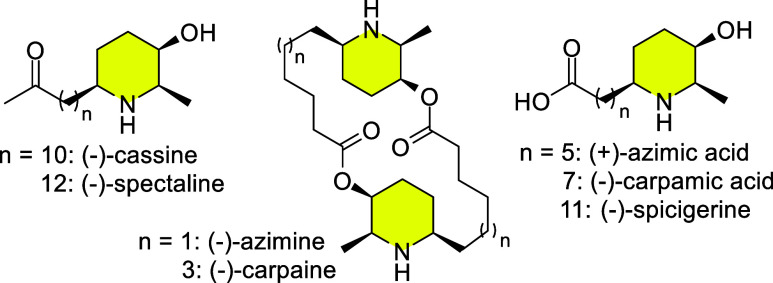
(−)-Cassine and other target piperidine alkaloids.

To date, seven stereoselective total syntheses of (−)-cassine and one of its enantiomer have been reported (Figure 2).^5^ The first asymmetric synthesis of (−)-cassine was disclosed by Momose. Starting from a meso diacetate already containing the piperidine ring (prepared in 10 steps from 1,5-cyclooctadiene), the authors were able to reach (−)-cassine after another 10 steps.^5a,5b^ Asymmetry was generated after a lipase-catalyzed hydrolysis of the meso diacetate. Meyer employed an enantiomerically pure β-hydroxyester (see Figure 2), obtained by lipase-catalyzed kinetic resolution, that after 10 steps furnished desired cassine.^5c^ Key steps involved the formation of a 2-oxazolidinone and its conversion to a key piperidin-3-ol (after hydrolysis, cyclic imine formation, and reduction). Hirota and co-workers^5d^ developed a 19-step linear synthesis starting from 1,5-hexadiyne, featuring a diastereoselective PdCl_2_-catalyzed cyclization as the key step. An interesting approach was described by Herdeis,^5e^ where the sugar l-rhamnose is employed as starting material. (−)-Cassine was obtained after 12 steps, involving 3.5 days transformation from a key azidoaldehyde and a phosphonate containing cassine’s long chain (HWE reaction, followed by addition of rhodium acetate). A short synthesis of (−)-cassine (7 steps from 11-bromo-1-undecanol) was accomplished by Kim^5f^ but with a cost of a low regioselectivity in the key asymmetric aminohydroxylation reaction. The latest total synthesis was described by Huang in 2010,^5g^ employing a reductive alkylation of lactams in 9 steps, starting from d-glutamic acid. Although low diastereoselectivity was observed in the key step (3.5:1), the authors managed to perform a step-economy synthesis, by performing several transformations in a one-pot reaction.

**Figure 2:**
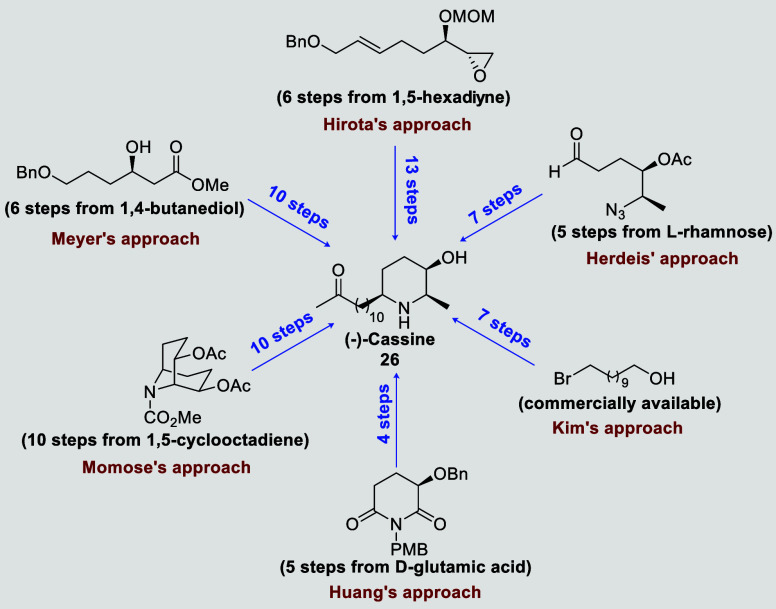
Previously reported synthetic strategies for (−)-cassine.

Despite the undeniable advances achieved over time to construct piperidine skeletons,^6^ as well as those described above for the total synthesis of cassine,^5^ some limitations still exist. For example, long linear approaches, strategies that do not allow access to synthetic analogues of the natural products, and low diastereoselective ratios on key steps are some examples. Therefore, the search for synthetic routes that encompass more modern principles of natural products total synthesis, such as convergent and divergent strategies, and the use of methodologies that provide products with high diastereoselectivity, becomes evident.^7^

Within this context, our research group developed two olefination reagents that allowed the selective synthesis of a wide range of α,β-unsaturated diazoketones, both with *E* and *Z* stereochemistry, with good yields, through a single Horner–Wadsworth–Emmons reaction (HWE).^8^ Because they are highly functionalized building blocks, containing a double bond and a carbonyl and a diazo group, the potential of α,β-unsaturated diazoketones in the synthesis of various alkaloids has been widely explored by our group over the years.^9^

Herein, we describe a new application of α,β-unsaturated diazoketones as useful synthetic tools for the asymmetric total synthesis of (−)-cassine in 10 steps from *N*-Cbz-*O*-TBDPS- *l* -serinal. To demonstrate the divergent character of this approach, different piperidine cores were synthesized using new alkylated diazophosphonates as olefination reagents.

Based on our previous experience on the total synthesis of nojirimycin skeletons,^9^ we envisaged that the piperidine core of (−)-cassine could be accessed through an N–H insertion reaction using the α,β-unsaturated diazoketone **6** (Scheme 1). In turn, this intermediate could be synthesized by our already established HWE protocol, using amino aldehyde **5** and novel methylated olefination reagent **1**. Next, the key cyclization to highly functionalized intermediate **9** will depend on a *cis*-selective N–H insertion reaction. Luche reduction followed by benzylation would provide allylic ether **24**. Finally, installation of the long aliphatic chain could be performed by a Wittig reaction, followed by a Wacker oxidation and a final deprotection/reduction step.

**Scheme 1:**
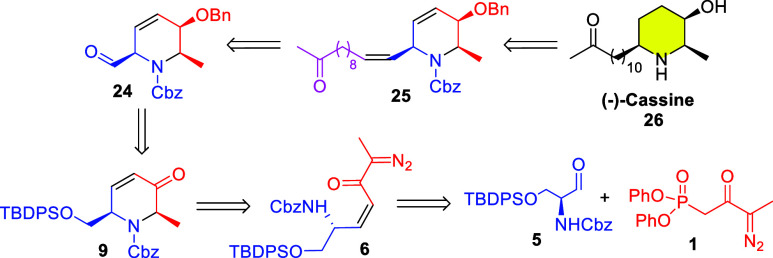
Proposed Retrosynthetic Route to (−)-Cassine

First, we focused our efforts on the synthesis of the novel diazophosphonate **1**. Following a similar approach developed by our research group,^8a^ this olefination reagent could be easily obtained from the reaction between diphenyl­(2-chloro-2-oxoethyl)­phosphonate and diazoethane. However, this reaction proved to be inefficient when diazomethane was replaced with diazoethane, providing the desired diazophosphonate **1** in only 12% yield along with a complex mixture of other products. The use of CaO as a chloride ion scavenger,^10^ distilled diazoethane, attempts to carry out larger scales, and reactions at lower temperatures also failed. Given these results, we decided to investigate a different strategy, promoting an alkylation of the already known diazophosphonate **2** using methyl iodide in the presence of a strong base.^11^ The results obtained are summarized in Table 1.

**Table 1:**
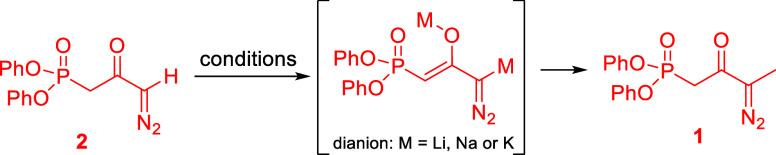
Studies on the Alkylation Reaction of **2**

entry^a^	base^e^	CH_3_I^e^ (eq)	temp	*t* (h)	yield
1^b^	*t*-BuOK (2.1)	2	–72 °C/0 °C	1/1	34%
2^b^	NaH (2.5)	2	–72 °C/0 °C	1/1	complex mixture
3^c^	KHMDS (2 + 2)	10	–72 °C to rt	16	complex mixture
4^b^	KHMDS (3.1)	2	–72 °C	2	45%
5^c^	LDA (2.1)	3	–72 °C	4	10%
6^c^	LDA (4)	2	–72 °C	1	6%
**7** ^b^	**LDA (2.1)**	**2**	**–72 °C**	**1**	**62%**
8^b^	LDA (2.1)	5	–72 °C	1	31%
9^d^	LDA (2.1)	2	–72 °C	1	47%

a All entries performed using dry THF (0.66 M) as solvent, except entry 3 (THF/HMPA, 1/1).

b Base added to a solution of diazophosphonate and methyl iodide.

c Diazophosphonate solution added to LDA, followed by methyl iodide.

d LDA added to a solution of diazophosphonate, followed by methyl iodide.

e Equivalent numbers.

No consumption of the starting material was observed with the use of *t*-BuOK as the base at −72 °C, for 1 h (entry 1). By increasing the temperature to 0 °C, after 60 min, the alkylated product was obtained in 34% yield. The use of NaH (entry 2) exhibited a similar profile at −72 °C, but at 0 °C, it provided a complex mixture of products. The use of KHMDS (3.1 equiv) (entry 4) provided the desired product in 45% yield. Next, LDA was evaluated as a base on different conditions (entries 5 to 9). The use of 2.1 equiv of LDA and 2 equiv of methyl iodide (entry 7) led to the best result (62%). It is also important to highlight that the order of addition of reagents proved to be of great influence on the reaction yield.

Given the good results obtained with this approach, we decided to evaluate the alkylation conditions of diazophosphonate **2** on other electrophiles (Scheme 2). Using benzyl iodide, the alkylated product **3** was obtained in 56% yield, while allyl bromide furnished diazophosphonate **4** in 45% yield. The use of different diazophosphonates in this synthetic route opens up a range of functionalization possibilities in the piperidine core, which reinforces the divergent approach of our strategy.

**Scheme 2:**
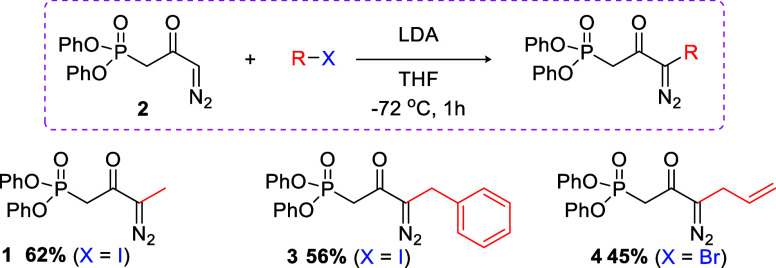
New Olefination Reagents Synthesized

The known building block *N*-Cbz-*O*-TBDPS-serinal **5** can be easily obtained in 5 steps from *l* -serine or in 3 steps from *N-Z-* *l* *-*serine methyl ester, both nonexpensive and commercially available starting materials.^9b^ Next, the HWE reaction could be evaluated using the three diazophosphonates prepared. Applying our protocol (Scheme 3), pure *Z*-α,β-unsaturated diazoketones **6**–**8** were obtained in 46–52% isolated yield after purification (diastereoselectivity = 5:1 for the reaction with **1** and 4:1 for **3**–**4**). This result was reproduced even on higher scales for product **6** (1.08 mmol, 500 mg of **5**). For the cyclization reaction, employing Cu­(acac)_2_ as the catalyst, the desired piperidine **9** was provided in 48% yield (the analogues **10** and **11** were obtained in 30% and 42% yield, respectively). Despite the moderate yield of this key step, it is important to mention the easy reaction setup, the short reaction time (1 min), and the fact that the product is obtained as a single *cis*-isomer. The stereochemical outcome was confirmed by NOE experiments on 3-piperidinone **9**. Interestingly, and in accordance with the observed NOE study, theoretical calculations revealed that the conformation with the two substituents at the pseudoaxial is the most stable one (Scheme 3, chart B; see Supporting Information for details).

**Scheme 3:**
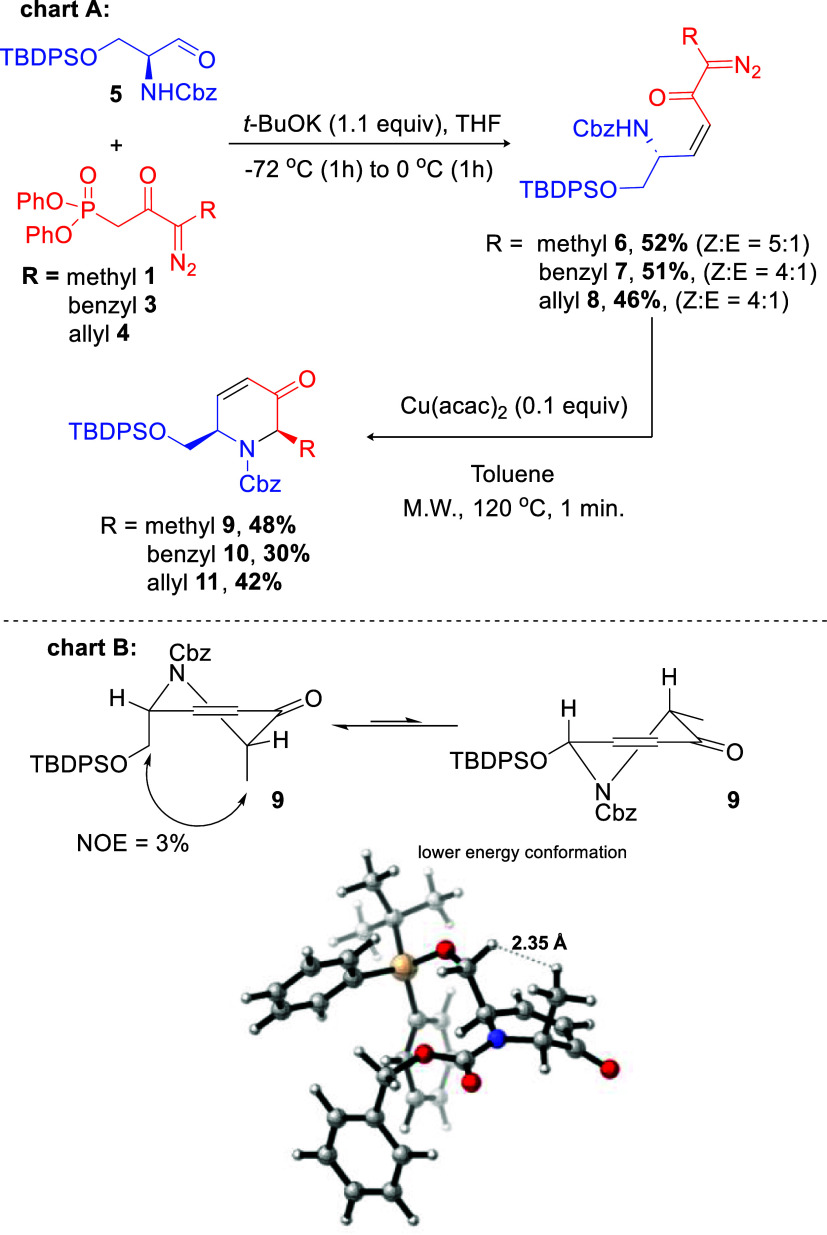
HWE and N–H Insertion Reactions Using New Alkylated Diazophosphonates

Aiming to reach the total synthesis of (−)-cassine, we next submitted enone **9** to a Luche reduction, providing the *cis*,*cis* allylic alcohol **12** in 92% yield (Scheme 4) as a single diastereoisomer (also confirmed by NOE experiments).

**Scheme 4:**
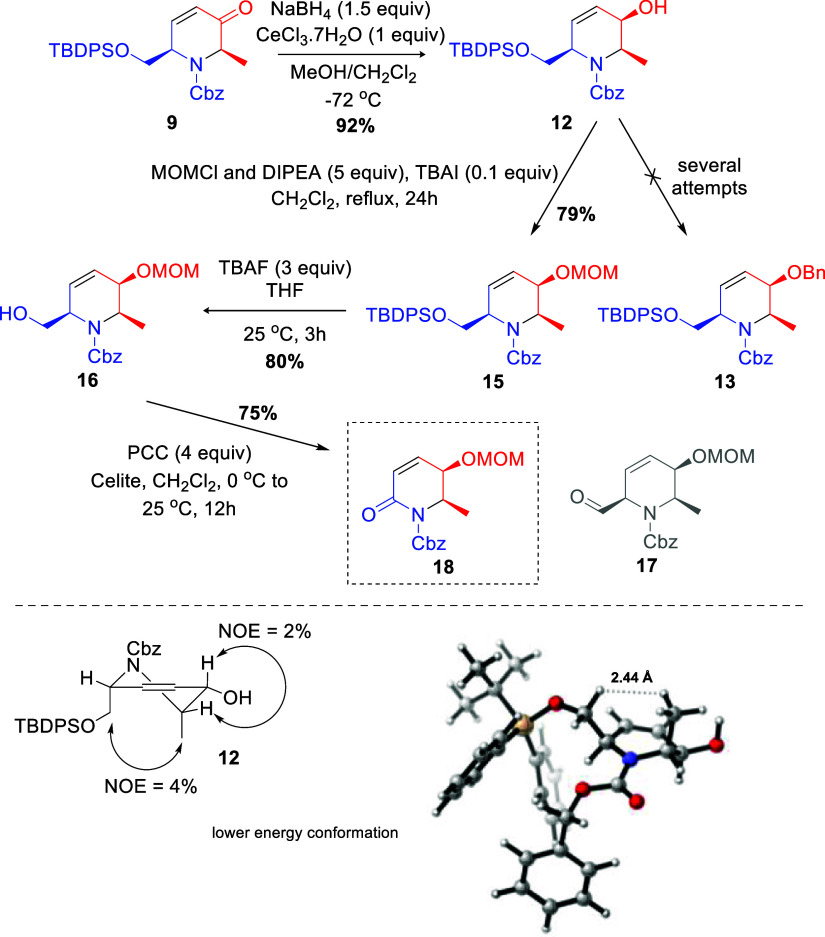
Studies on the Total Synthesis of (−)-Cassine

From this point, and as illustrated in Scheme 1, installation of a benzyl protecting group for the hydroxyl group in **12** would be the best choice since it could be removed in a single step at the end of the route, together with the Cbz group and the reduction of the endocyclic double bond. To our surprise, poor results were obtained after evaluation of several benzylation conditions (Table S1, see SI). Even benzyl trichloroacetimidate, a powerful benzylation reagent,^12^ failed to provide product **13**. As a consequence, other protecting groups were evaluated. No reaction occurred using benzyl chloroformate, but good yields were obtained using *p*-nitrobenzoyl chloride (**14**, 65% yield; see Supporting Information) and chloromethyl methyl ether (**15**, 79% yield). We decided to move forward using the MOM group, as it furnished the best yield. The TBDPS deprotection of **15** using tetrabutylammonium fluoride (TBAF, 3 equiv) in THF proceeded smoothly, furnishing the alcohol **16** in 80% yield. The following steps consisted of the oxidation to the corresponding aldehyde and the coupling of the long aliphatic chain via Wittig olefination. Unfortunately, several oxidation methods failed to give the aldehyde **17**, with results varying from complex mixtures to no consumption of the starting material (Table S2, see the SI). Interestingly, the use of PCC for 12 h at room temperature resulted in the decarboxylated product **18** in 75% yield (Scheme 4). Based on this result, we considered that the presence of the double bond of the piperidine core may have an influence on this step, leading to undesired product **18**. To put this hypothesis to the test, the olefin was reduced with a hydrogen atmosphere and PtO_2_ (0.1 equiv) and the hydrogenated product **19** was submitted to oxidation conditions with IBX (Scheme 5). After this sequence, aldehyde **20** was obtained in 94% yield.

**Scheme 5:**
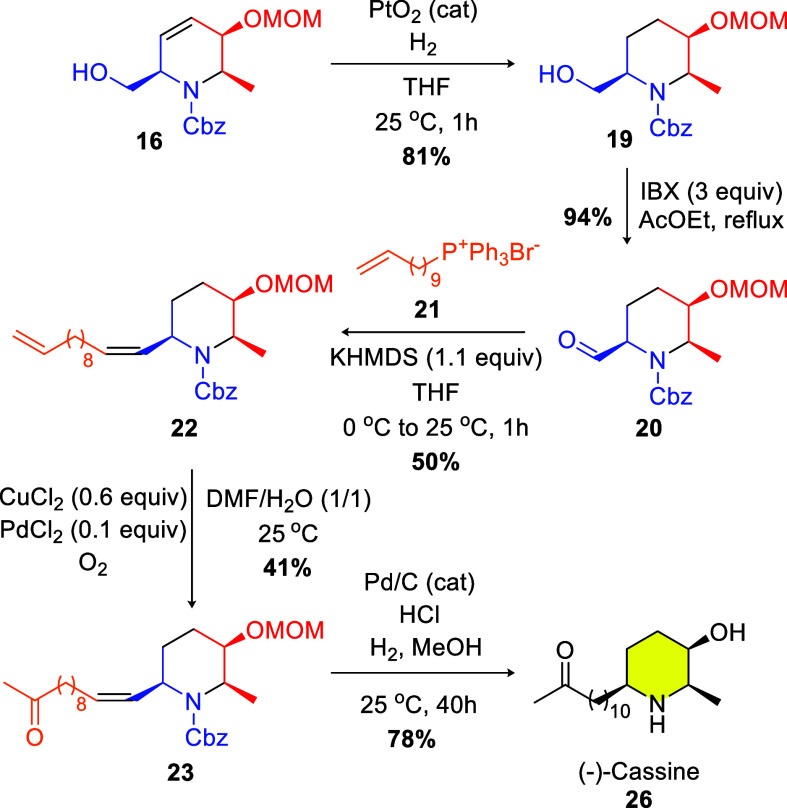
Last Steps of the Synthetic Route

Next, the phosphonium salt **21**, easily prepared from commercially available 11-bromo-1-undecene following literature procedures,^13^ was submitted to aldehyde **20**, in the presence of KHMDS. The Wittig olefination product **22** was obtained in 50% yield (Scheme 5). Making use of the conditions described by Hirota and co-workers,^5d^ the Wacker oxidation proceeded well, furnishing the methyl ketone **23** in 41% yield.

For the last step of the total synthesis, substrate **23** was treated with Pd/C and hydrogen to remove the Cbz group and to reduce the exocyclic double bond, previously formed in the Wittig reaction. After 16 h, the reaction vessel was opened and a few drops of concentrated HCl were added for MOM group removal. The reaction ended after 24 h and standard workup procedures furnished (−)-cassine in 78% yield. The spectroscopic data were in agreement with those already reported in the literature (Tables S3 and S4, see SI).^5g^

## Conclusions

In summary, the asymmetric total synthesis of (−)-cassine was accomplished in 10 steps from *N*-Cbz-*O*-TBDPS-serinal. Key steps involved a complete diastereoselective intramolecular N–H insertion reaction and Luche reduction, leading to a single *cis*,*cis*-isomer. Three new olefination reagents were synthesized from diazophosphonate **2** using an alkylation strategy and were converted to three novel highly functionalized intermediates. With different substituents on the piperidine core, these intermediates can be applied not only to the synthesis of cassine and its analogues but also to other piperidine alkaloids. This divergent approach reinforces the versatility and applicability of α,β-unsaturated diazoketones in the total synthesis of natural products and analogues.

## Experimental Section

**Caution!** *o*-Iodoxybenzoic acid (IBX) is an oxidizer and may pose a risk of explosion under certain conditions, especially when it is dry or finely powdered. It should be handled in small quantities and stored in a tightly sealed container away from heat or friction.

**Caution!** *n*-Butyllithium (*n*-BuLi) is a pyrophoric reagent, reacts violently with water, and must be handled under an inert atmosphere using air-free techniques.

**Caution!** Hydrogen is classified as a GHS Flammable Gas in Category 1. A hydrogen balloon was handled in a fume hood with exhaust and away from flammable materials.

Diazophosphonates **1**, **2**, **3**, and **4** can be stored in a refrigerator at 4 °C for several months without degradation and did not exhibit explosive behavior during the course of this work.

### General Procedures

All solvents were dried and distilled prior to use by standard procedures. Reagents and metal catalysts were used without further purification unless stated otherwise. Reactions were monitored by thin layer chromatography (TLC), carried out using silica gel 60 F254 precoated plates, with UV light (254 nm) as the visualizing agent and potassium permanganate in aqueous KOH for staining. Flash column chromatography was performed using silica gel 60 (200–400 Mesh). Unless stated otherwise, all yields refer to isolated products after purification by flash column chromatography. The solvent mixtures employed in TLC analysis and in flash column chromatography purifications are reported as volumes per volume and in percentages. Proton nuclear magnetic resonance (^1^H NMR), carbon nuclear magnetic resonance (^13^C­{^1^H} NMR), and NOE (Nuclear Overhauser Effect) spectra were recorded using an Agilent Technologies −500/54 Premium Shielded (500 MHz for ^1^H NMR and 125 MHz for ^13^C­{H} NMR) instrument. For ^1^H NMR spectra, chemical shifts are referenced from TMS (0.00 ppm) or DMSO-*d*_6_ (solvent residual peak at 2.5 ppm). Coupling constants (J) are reported in Hz. For the ^13^C­{^1^H} NMR spectra, chemical shifts are referenced from CDCl_3_ (77.16 ppm) or DMSO-*d*_6_ (39.5 ppm). High-resolution mass spectra (HRMS) were recorded using electron spray ionization (ESI) (QqTOF/MS-MicrOTOF-Q II models). Optical rotations were recorded on a Jasco Digital P-2000 Polarimeter with a 1 dm cell and a sodium lamp.

### Preparation of Lithium Diisopropylamide (LDA)

To a flame-dried 10 mL round-bottom flask under an argon atmosphere were added anhydrous THF (3.4 mL) and diisopropylamine (460 μL, 3.3 mmol). The solution was cooled to −72 °C and *n*-BuLi was added (1.5 mL, 2.2 M in hexanes, 3.3 mmol, 1 equiv). The reaction mixture was stirred at −72 °C for 10 min before use.

### General Procedure for the Alkylation Reaction of Diazophosphonate (**2)**

To a round-bottom flask containing diphenyl (3-diazo-2-oxopropyl) phosphonate **2** (1.58 mmol, 1 equiv) under an argon atmosphere were added anhydrous THF (0.66 M) and the alkylating reagent (3.16 mmol, 2.0 equiv). The solution was cooled to −72 °C and freshly prepared LDA was added dropwise (3.32 mmol, 2.1 equiv). After 1 h of stirring at −72 °C, the reaction was quenched with saturated aqueous NH_4_Cl solution. The aqueous layer was extracted three times with ethyl acetate, and the combined layers were washed with brine, dried over anhydrous Na_2_SO_4_, and filtered. The solvent was removed under reduced pressure to furnish the crude product that was purified by flash column chromatography (silica gel, *n*-hexane/diethyl ether 1:1 or hexane/ethyl acetate 7:3) to obtain the alkylated diazophosphonate.

### Diphenyl­(3-diazo-2-oxobutyl)­phosphonate **(1)**

Prepared using iodomethane as the alkylating reagent: 62% yield (323 mg), yellow oil, *R*_*f*_ = 0.29 (*n*-hexane:ethyl acetate, 1:1); ^1^H NMR (500 MHz, CDCl_3_, 26 °C): δ 7.37–7.32 (m, 4H), 7.25–7.18 (m, 6H), 3.46/3.37 (two doublets of two inseparable conformers, *J* = 22.3/22.6 Hz, 2H), 2.22/2.01 (two singlets of two inseparable conformers, 3H); ^13^C­{^1^H} NMR (126 MHz, CDCl_3_, 26 °C): δ 182.8 (d, *J* = 6.6 Hz), 150.0 (d, *J* = 8.8 Hz), 129.8 (d, *J* = 1.1 Hz), 125.6 (d, *J* = 1.2 Hz), 120.6 (d, *J* = 4.4 Hz), 65.6, 37.3 (d, *J* = 133.0 Hz), 10.1 (minority conformer), 8.5; IR: ν_max_ (neat, ATR) 3066, 2980, 2927, 2080, 1616, 1591, 1488, 1276, 1186, 1025, 930, 764 cm-1; HRMS (ESI + qTOF) *m*/*z*: [M + Na]^+^ calcd for C_16_H_15_N_2_NaO_4_P^+^ 353.0662; found, 353.0661.

### Diphenyl­(3-diazo-2-oxo-4-phenylbutyl)­phosphonate **(3)**

Prepared using benzyl iodide as the alkylating reagent: 56% yield (360 mg), yellow oil, *R*_*f*_ = 0.27 (*n*-hexane:ethyl acetate, 7:3); ^1^H NMR (500 MHz, CDCl_3_, 26 °C): δ 7.36–7.14 (m, 15H), 3.91/3.72 (two singlets of two inseparable conformers, 2H), 3.47/3.31 (two doublets of two inseparable conformers, *J* = 22.2/22.4 Hz, 2H); ^13^C­{^1^H} NMR (126 MHz, CDCl_3_, 26 °C): δ 181.8 (d, *J* = 6.6 Hz), 149.9 (d, *J* = 8.8 Hz), 136.3 (s), 129.8 (s), 128.9 (s), 128.5 (s), 127.3 (s), 125.6 (d, *J* = 1.1 Hz), 120.6 (d, *J* = 4.4 Hz), 71.0, 37.6 (d, *J* = 132.5 Hz), 30.4 (minority conformer), 29.0; IR: ν_max_ (neat, ATR) 3063, 3029, 2924, 2078, 1624, 1589, 1487, 1352, 1275, 1209, 1182, 1160, 925, 903, 758, 687 cm^–1^; HRMS (ESI + qTOF) *m*/*z*: [M + Na]^+^ calcd for C_22_H_19_N_2_NaO_4_P^+^ 429.0975; found, 429.0976.

### Diphenyl­(3-diazo-2-oxohex-5-en-1-yl)­phosphonate **(4)**

Prepared using allyl bromide as the alkylating reagent: 45% yield (253 mg), yellow oil, *R*_*f*_ = 0.36 (*n*-hexane:ethyl acetate, 6:4); ^1^H NMR (500 MHz, CDCl_3_, 26 °C): δ 7.36–7.31 (m, 4H), 7.25–7.17 (m, 6H), 5.90–5.82 and 5.77 (m and ddt of two inseparable conformers, *J* = 16.7, 10.0, 6.5 Hz, 1H), 5.20 (dd, *J* = 2.8, 1.5 Hz, 1H), 5.17 (ddd, *J* = 5.3, 3.3, 0.9 Hz, 1H), 3.47/3.36 (two doublets of two inseparable conformers, *J* = 22.2/22.4 Hz, 2H), 3.29/3.14 (two doublets of two inseparable conformers, *J* = 5.4/6.4 Hz, 2H); ^13^C­{^1^H} NMR (126 MHz, CDCl_3_, 26 °C): δ 181.7 (d, *J* = 6.5 Hz), 149.9 (d, *J* = 8.8 Hz), 131.5 (s), 129.8 (d, *J* = 0.8 Hz), 125.6 (d, *J* = 1.2 Hz), 120.6 (d, *J* = 4.4 Hz), 118.4, 69.1 (s), 38.1, 37.8 (minority conformer), 37.1, 36.7 (minority conformer), 28.7, 26.9; IR: ν_max_ (neat, ATR) 3069, 2978, 2924, 2079, 1623, 1590, 1488, 1348, 1275, 1209, 1182, 1161, 924, 760, 688 cm^–1^; HRMS (ESI + qTOF) *m*/*z*: [M + Na]^+^ calcd for C_18_H_17_N_2_NaO_4_P^+^ 379.0818; found, 379.0819.

### General Procedure for the HWE Reaction

To a round-bottom flask containing *t*-BuOK (1.1 mmol, 1.1 equiv) under an argon atmosphere was added anhydrous THF (0.3 M). The suspension was cooled to 0 °C and a solution of the alkylated diazophosphonate (1 mmol, 1.0 equiv) in dry THF (0.05 M) was added. After 10 min, the solution was cooled to −72 °C and a solution of **5** (1 mmol, 1.0 equiv) in dry THF (0.2 M) was added. After 1 h, the temperature was immediately allowed to increase to 0 °C and the mixture was stirred for an additional 1 h. Then, a saturated aqueous NH_4_Cl solution was added to the reaction vessel. The aqueous layer was extracted three times with ethyl acetate, and the combined organic layers were washed with brine, dried over Na_2_SO_4_, and filtered. The solvent was removed under reduced pressure to furnish the crude product that was purified by flash column chromatography (silica gel, 4:1 *n*-hexane/ethyl acetate) to give *Z*-α,β-unsaturated diazoketone and the *E* isomer.

### (R,Z)-Benzyl (1-((*tert*-butyldiphenylsilyl)­oxy)-6-diazo-5-oxohept-3-en-2-yl)­carbamate (**6)**

Yellow oil, 52% yield for *Z* isomer (282 mg); (*Z:E* ratio (isolated) = 5:1); *R*_*f*_ = 0.4 (*n*-hexane:ethyl acetate, 7:3); ^1^H NMR (500 MHz, DMSO-*d*_6_, 80 °C): δ 7.68–7.55 (m, 4H), 7.48–7.26 (m, 11H), 7.13 (s, 1H), 6.36 (d, *J* = 11.6 Hz, 1H), 6.01 (dd, *J* = 11.6, 8.7 Hz, 1H), 5.27–5.17 (m, 1H), 5.03 (s, 2H), 3.77–3.68 (m, 2H), 1.94 (s, 3H), 1.00 (s, 9H); ^13^C­{^1^H} NMR (126 MHz, DMSO-*d*_6_, 80 °C): δ 184.8, 155.1, 141.3, 136.8, 134.7, 132.9, 132.9, 129.2, 127.8, 127.2, 127.2, 127.1, 123.6, 65.2, 65.0, 63.5, 51.1, 26.2, 18.5, 7.6; IR: ν_max_ (neat, ATR) 3438, 3334, 3070, 2955, 2930, 2857, 2062, 1715, 1642, 1602, 1498, 1427, 1318, 1283, 1236, 1108, 1048, 608 cm^–1^; [α_D_]^25^ = −28,54 (*c* = 3.5, CHCl_3_); HRMS (ESI + qTOF) *m*/*z*: [M + Na]^+^ calcd for C_31_H_35_N_3_NaO_4_Si^+^ 564.2289; found, 564.2295.

### (R,Z)-Benzyl (1-((*tert*-butyldiphenylsilyl)­oxy)-6-diazo-5-oxo-7-phenylhept-3-en-2-yl)­carbamate **(7)**

Yellow oil, 51% yield for *Z* isomer (276 mg); (*Z:E* ratio (isolated) = 4:1); *R*_*f*_ = 0.45 (*n*-hexane:ethyl acetate, 7:3) ^1^H NMR (500 MHz, DMSO-*d*_6_, 80 °C): δ 7.66–7.60 (m, 4H), 7.46–7.28 (m, 15H), 7.26–7.22 (m, 2H), 6.41 (d, *J* = 11.6 Hz, 1H), 6.04 (dd, *J* = 11.5, 8.7 Hz, 1H), 5.31–5.23 (m, 1H), 5.04 (d, *J* = 1.3 Hz, 2H), 3.77–3.66 (m, 4H), 1.00 (s, 9H); ^13^C­{^1^H} NMR (126 MHz, DMSO-*d*_6_, 80 °C): δ 196.3, 155.1, 142.0, 136.8, 136.8, 134.7, 132.9, 132.9, 129.2, 128.2, 127.8, 127.8, 127.3, 127.3, 127.2, 127.1, 126.4, 123.6, 68.8, 65.2, 65.0, 51.2, 26.3, 18.5; IR: ν_max_ (neat, ATR) 3437, 3336, 3068, 3031, 2955, 2931, 2889, 2857, 2067, 1720, 1603, 1497, 1427, 1323, 1236, 1112, 1076, 701 cm^–1^; [α_D_]^25^ = −6.66 (*c* = 0.3, CHCl3). HRMS (ESI + qTOF) *m*/*z*: [M + Na]^+^ calcd for C_37_H_39_N_3_O_4_NaSi^+^ 640.2602; found, 640.2604.

### (R,Z)-Benzyl (1-((*tert*-butyldiphenylsilyl)­oxy)-6-diazo-5-oxonona-3,8-dien-2-yl)­carbamate (**8)**

Yellow oil, 46% yield for *Z* isomer (261 mg); *Z:E* ratio (isolated) = 4:1); *R*_*f*_ = 0.4 (*n*-hexane:ethyl acetate, 4:1); ^1^H NMR (500 MHz, DMSO-*d*_6_, 80 °C): δ 7.68–7.54 (m, 4H), 7.49–7.24 (m, 11H), 7.17 (s, 1H), 6.38 (d, *J* = 11.6 Hz, 1H), 6.03 (dd, *J* = 11.6, 8.7 Hz, 1H), 5.81 (ddt, *J* = 16.5, 10.1, 6.3 Hz, 1H), 5.27–5.19 (m, 1H), 5.14 (ddd, *J* = 13.6, 11.2, 1.3 Hz, 2H), 5.03 (s, 2H), 3.76–3.68 (m, 2H), 3.11 (d, *J* = 6.0 Hz, 2H, superimposed with H_2_O), 1.00 (s, 9H); ^13^C­{^1^H} NMR (126 MHz, DMSO-*d*_6_, 80 °C): δ 155.1, 141.7, 136.8, 134.7, 132.9, 132.4, 129.4, 129.2, 127.9, 127.8, 127.3, 127.2, 127.1, 123.7, 117.1, 65.2, 65.0, 58.4, 51.1, 26.3, 26.3, 18.5; IR: ν_max_ (neat, ATR) 3431, 3338, 3070, 2955, 2931, 2857, 2066, 1716, 1499, 1427, 1258, 1235, 1110, 1086, 740, 700 cm^–1^; [α_D_]^25^ = −19 (*c* = 1, CHCl_3_). HRMS (ESI + qTOF) *m*/*z*: [M + Na]^+^ calcd for C_33_H_37_N_3_O_4_NaSi^+^ 590.2445; found, 590.2453.

### General Procedure for the N–H Insertion Reaction

To a microwave reaction vessel (6 mL capacity) were added the *Z*-α,β-unsaturated diazoketone (0.277 mmol, 1 equiv), toluene (0.05 M), and copper­(II) acetylacetonate (27.7 μmol, 0.1 equiv). The reaction vessel was sealed. Using an Anton Paar microwave reactor (Monowave 300) equipped with a ruby sensor for internal temperature monitoring, the reaction mixture was heated to 120 °C over 2 min and maintained at this temperature with vigorous stirring for 1 min (2 bar pressure). After cooling to 45 °C, the solvent was removed under reduced pressure and the crude product was purified by flash column chromatography (silica gel, 3:2 *n*-hexane/diethyl ether) to furnish the cyclized product as a single diastereoisomer.

### Benzyl­(2*R*,6*R*)-6-(((*tert*-butyldiphenylsilyl)­oxy)­methyl)-2-methyl-3-oxo-3,6-dihydropyridine-1­(2H)-carboxylate **(9)**

Colorless oil, 48% yield (68 mg), *R*_*f*_ = 0.45 (*n*-hexane:ethyl acetate, 4:1); ^1^H NMR (500 MHz, DMSO-*d*_6_, 80 °C): δ 7.60 (m, 4H), 7.46 (m, 2H), 7.41 (m, 4H), 7.33 (br s, 5H), 7.22 (dd, *J* = 10.5, 4.7 Hz, 1H), 6.17 (dd, *J* = 10.5, 1.8 Hz, 1H), 5.12 (dd, *J* = 31.1, 12.5 Hz, 2H), 4.94 (td, *J* = 6.3, 1.8 Hz, 1H), 4.54 (q, *J* = 7.2 Hz, 1H), 3.91–3.83 (m, 2H), 1.26 (d, *J* = 7.2 Hz, 3H), 1.01 (s, 9H); ^13^C­{^1^H} NMR (126 MHz, DMSO-*d*_6_, 80 °C): δ 194.4, 153.9, 147.6, 136.0, 134.7, 134.6, 132.3, 132.1, 129.5, 129.5, 127.9, 127.5, 127.4, 127.4, 127.2, 125.2, 66.5, 65.0, 55.3, 53.3, 26.3, 19.4, 18.4; IR: ν_max_ (neat, ATR) 3070, 2956, 2931, 2858, 1688, 1427, 1320, 1307, 1285, 1110, 1045, 701 cm^–1^; [α_D_]^25^ = +75.42 (*c* = 4.5, CHCl_3_); HRMS (ESI + qTOF) *m*/*z*: [M + H]^+^ calcd for C_31_H_36_NO_4_Si^+^ 514.2408; found, 514.2414.

### (2*R*,6*R*)-Benzyl-6-benzyl-2-(((*tert*-butyldiphenylsilyl)­oxy)­methyl)-5-oxo-5,6-dihydropyridine-1­(2H)-carboxylate **(10)**

Colorless oil, 30% yield (49 mg), *R*_*f*_ = 0.39 (*n*-hexane:ethyl acetate, 4:1); ^1^H NMR (500 MHz, DMSO-*d*_6_, 80 °C): δ 7.65–7.55 (m, 4H), 7.50–7.41 (m, 6H), 7.34–7.30 (m, 3H), 7.25–7.20 (m, 3H), 7.12–7.07 (m, 3H), 6.96–6.91 (m, 2H), 6.19 (dd, *J* = 10.5, 2.0 Hz, 1H), 5.05 (d, *J* = 12.4 Hz, 1H), 4.96–4.86 (m, 2H), 4.74 (t, *J* = 6.3 Hz, 1H), 3.87 (s, 1H), 3.59 (s, 1H), 2.94–2.86 (m, 2H), 1.03 (s, 9H); ^13^C­{^1^H} NMR (126 MHz, DMSO-*d*_6_ 80 °C): δ 192.7, 154.1, 147.4, 136.5, 135.8, 134.7, 134.7, 132.4, 132.2, 129.5, 129.5, 128.7, 127.9, 127.6, 127.5, 127.4, 127.3, 126.0, 125.1, 66.6, 65.1, 60.7, 53.6, 26.4, 18.4; IR: ν_max_ (neat, ATR) 3069, 3031, 2955, 2931, 2889, 2857, 1697, 1683, 1414, 1305, 1279, 1248, 1104, 1050, 823, 739, 690 cm^–1^; [α_D_]^25^ = +31,2 (*c* = 1, CHCl_3_) HRMS (ESI + qTOF) *m*/*z*: [M + H]^+^ calcd for C_37_H_40_NO_4_Si^+^ 590.2721; found, 590.2726.

### (2*R*,6*R*)-Benzyl-6-allyl-2-(((*tert*-butyldiphenylsilyl)­oxy) methyl)-5-oxo-5,6-dihydropyridine-1­(2H)-carboxylate **(11)**

Colorless oil, 42% yield (63 mg), *R*_*f*_ = 0.47 (*n*-hexane:ethyl acetate, 4:1); ^1^H NMR (500 MHz, DMSO-*d*_6_, 80 °C): δ 7.64–7.58 (m, 4H), 7.50–7.38 (m, 6H), 7.32 (br s, 5H), 7.24 (dd, *J* = 10.5, 4.4 Hz, 1H), 6.16 (dd, *J* = 10.5, 1.8 Hz, 1H), 5.60 (ddt, *J* = 17.1, 10.2, 7.1 Hz, 1H), 5.10 (d, *J* = 3.8 Hz, 2H), 4.93 (ddd, *J* = 9.6, 7.1, 2.1 Hz, 1H), 4.86–4.75 (m, 2H), 4.57 (t, *J* = 7.4 Hz, 1H), 3.92 (dd, *J* = 9.7, 5.3 Hz, 1H), 3.80 (dd, *J* = 9.4, 7.8 Hz, 1H), 2.39 (dt, *J* = 13.3, 6.6 Hz, 1H), 2.28–2.20 (m, 1H), 1.03 (s, 9H); ^13^C­{^1^H} NMR (126 MHz, DMSO-*d*_6_, 80 °C): δ 192.9, 154.1, 147.4, 135.9, 134.7, 134.6, 134.1, 133.3, 132.3, 132.2, 129.5, 129.5, 127.9, 127.5, 127.4, 127.4, 127.2, 125.0, 116.9, 66.6, 65.1, 58.9, 53.4, 38.6, 26.3, 18.4; IR: ν_max_ (neat, ATR) 3071, 3047, 3033, 2958, 2931, 2890, 2857, 1687, 1427, 1414, 1389, 1306, 1247, 1105, 1049, 998, 823, 741, 700 cm^–1^; [α_D_]^25^ = +30.69 (*c* = 1.3, CHCl_3_). HRMS (ESI + qTOF) *m*/*z*: [M + Na]^+^ calcd for C_33_H_37_NO_4_NaSi^+^ 562.2384; found, 562.2391.

### Procedure for the Luche Reduction (**12**)

Cerium trichloride heptahydrate (153 mg, 0.41 mmol, 1 equiv) was dissolved in anhydrous methanol (1 mL, 0.4 M) and this solution was added in a 25 mL round-bottom flask containing a solution of dihydropyridin-3-one **9** (212 mg, 0.41 mmol, 1 equiv) in dichloromethane (6.8 mL, 0.06 M) at −72 °C. After 20 min, sodium borohydride (23.2 mg, 0.61 mmol, 1.5 equiv) was added, and the resulting solution was stirred for 1 h. After this period, the reaction mixture was warmed to 25 °C and the reaction quenched with water. The layers were separated and the aqueous portion was extracted with ethyl acetate (3 × 15 mL). The combined organic layers were dried over anhydrous Na_2_SO_4_ and concentrated under reduced pressure. The crude product was purified by flash column chromatography (silica gel, 7:3 *n*-hexane/ethyl acetate) to furnish the allylic alcohol **12** as a colorless oil: 92% yield (194 mg), *R*_*f*_ = 0.32 (*n*-hexane:ethyl acetate, 7:3); ^1^H NMR (500 MHz, DMSO-*d*_6_, 80 °C): δ 7.61–7.56 (m, 4H), 7.46–7.35 (m, 6H), 7.32–7.24 (m, 5H), 5.90 (dt, *J* = 10.5, 2.8 Hz, 1H), 5.70–5.65 (m, 1H), 5.07 (d, *J* = 12.7 Hz, 1H), 5.03 (d, *J* = 12.7 Hz, 1H), 4.95 (br s, 1H), 4.43 (q, *J* = 6.7 Hz, 1H), 4.41–4.36 (m, 1H), 4.20 (m, 1H), 3.81 (dd, *J* = 9.0, 5.0 Hz, 1H), 3.58 (t, *J* = 8.9 Hz, 1H), 1.00 (s, 9H), 0.86 (d, *J* = 6.9 Hz, 3H); ^13^C­{^1^H} NMR (126 MHz, DMSO-*d*_6_, 80 °C): δ 154.1, 136.4, 134.6, 134.6, 132.6, 132.6, 129.4 (2x), 129.1, 128.0, 127.4, 127.3, 127.0, 123.0, 66.0, 64.8, 64.7, 52.4, 47.9, 26.3, 18.4, 13.4; IR: ν_max_ (neat, ATR) 3441, 3070, 3035, 2956, 2931, 2888, 2857, 1679, 1414, 1300, 1107, 1081, 1023, 732, 698 cm^–1^; [α_D_]^25^ = +62.47 (*c* = 1.7, CHCl_3_); HRMS (ESI + qTOF) *m*/*z*: [M + H]^+^ calcd for C_31_H_38_NO_4_Si^+^ 516.2565; found, 516.2574.

### Procedure for the MOM Protection (**15**)

To a 125 mL two-neck round-bottom flask equipped with a reflux condenser containing the allylic alcohol **12** (705 mg, 1.37 mmol, 1 equiv) under an argon atmosphere were added anhydrous dichloromethane (0.02 M), MOMCl (502 μL, 6.85 mmol, 5 equiv), and DIPEA (1.2 mL, 6.85 mmol, 5 equiv). The system was open briefly and TBAI was added (51.5 mg, 0.137 mmol, 0.1 equiv). The reaction mixture was refluxed over 22 h using an oil bath. Then, water was added, the layers were separated, and the aqueous portion was extracted with dichloromethane (3 × 25 mL). The combined organic layers were washed with brine, dried over anhydrous Na_2_SO_4_, and concentrated under reduced pressure. The crude product was purified by flash column chromatography (silica gel, 6:4 *n*-hexane:diethyl ether) to furnish the protected allylic alcohol **15** as a colorless oil: 79% yield (606 mg), *R*_*f*_ = 0.38 (*n*-hexane:diethyl ether, 7:3); ^1^H NMR (500 MHz, DMSO-*d*_6_, 80 °C): δ 7.63–7.58 (m, 4H), 7.48–7.37 (m, 6H), 7.33–7.26 (m, 5H), 5.99 (dt, *J* = 10.6, 2.7 Hz, 1H), 5.77 (d, *J* = 10.6 Hz, 1H), 5.10 (d, *J* = 12.6 Hz, 1H), 5.06 (d, *J* = 12.7 Hz, 1H), 4.69 (d, *J* = 6.6 Hz, 1H), 4.67 (d, *J* = 6.7 Hz, 1H), 4.61 (p, *J* = 6.5 Hz, 1H), 4.46–4.40 (m, 1H), 4.22–4.18 (m, 1H), 3.84 (dd, *J* = 9.2, 5.1 Hz, 1H), 3.62 (t, *J* = 9.0 Hz, 1H), 3.31 (s, 3H), 1.02 (s, 9H), 0.92 (d, *J* = 6.9 Hz, 3H); ^13^C­{^1^H} NMR (126 MHz, DMSO-*d*_6_, 80 °C): δ 154.1, 136.3, 134.6, 134.6, 132.6, 132.5, 129.4, 129.4, 127.9, 127.4, 127.3, 126.9, 126.4, 124.3, 95.2, 71.3, 66.1, 64.7, 54.7, 52.6, 46.1, 26.3, 18.4, 13.9; IR: ν_max_ (neat, ATR) 3070, 3048, 2950, 2932, 2887, 2857, 1698, 1413, 1299, 1150, 1102, 1040, 1006, 982, 917, 823, 768, 737, 697 cm^–1^; [α_D_]^25^ = +57.33 (*c* = 2.4, CHCl_3_); HRMS (ESI + qTOF) *m*/*z*: [M + H]^+^ calcd for C_33_H_42_NO_5_Si^+^ 560.2827; found, 560.2826.

### Procedure for the TBDPS Deprotection (**16**)

To a 25 mL round-bottom flask were added the MOM-protected alcohol **15** (177 mg, 0.316 mmol, 1 equiv), anhydrous THF (16 mL, 0.02 M), and TBAF (0.95 mL, 1.0 M in THF, 3 equiv) at 25 °C. The reaction mixture was stirred for 90 min at room temperature. After this time, the solvent was removed under reduced pressure. Ethyl acetate was added (15 mL) and the organic phase was washed with water (2 × 10 mL) and brine (1 × 10 mL). The desired product was used in the next step without further purification: 80% yield (81 mg), *R*_*f*_ = 0.19 (*n*-hexane:ethyl acetate, 3:2); ^1^H NMR (400 MHz, CDCl_3_, 26 °C): δ 7.41–7.30 (m, 5H), 5.84–5.72 (m, 2H), 5.19 (s, 2H), 4.71 (q, *J* = 6.8 Hz, 3H), 4.47 (s, 1H), 4.28 (s, 1H), 3.75 (t, *J* = 8.3 Hz, 1H), 3.67 (s, 1H), 3.40 (s, 3H), 1.11 (d, *J* = 6.9 Hz, 3H); ^13^C­{^1^H} NMR (101 MHz, CDCl_3_, 26 °C): δ 156.9, 136.3, 128.6, 128.2, 127.8, 127.1, 124.5, 95.8, 71.6, 67.6, 66.2, 55.7, 54.4, 46.9, 14.6; IR: ν_max_ (neat, ATR) 3450, 2941, 2888, 1693, 1677, 1414, 1314, 1149, 1104, 1028, 1004, 981, 917, 698 cm^–1^; [α_D_]^25^ = +66.61 (*c* = 1.3, CHCl_3_); HRMS (ESI + qTOF) *m*/*z*: [M + Na]^+^ calcd for C_17_H_23_NNaO_5_^+^ 344.1468; found, 344.1465.

### Procedure for the Double Bond Reduction (**19**)

To a 5 mL round-bottom flask containing the olefin **16** (20 mg, 0.062 mmol, 1 equiv) were added anhydrous THF (2 mL, 0.03 M) and PtO_2_ (1.43 mg, 6.2 μmol, 0.1 equiv). Hydrogen gas was bubbled in the solution, and the reaction mixture was stirred under a hydrogen atmosphere at room temperature for 1 h. Then, the reaction mixture was diluted with THF (2 mL) and filtered through a small plug of Celite. The solvent was removed under reduced pressure and the hydrogenated product **19** was obtained in 81% yield (16 mg) as a colorless oil without further purification. *R*_*f*_ = 0.3 (*n*-hexane:ethyl acetate, 1:1); ^1^H NMR (400 MHz, CDCl_3_, 26 °C): δ 7.40–7.28 (m, 5H), 5.19 (d, *J* = 12.4 Hz, 1H), 5.13 (d, *J* = 12.4 Hz, 1H), 4.66 (s, 2H), 4.51 (p, *J* = 6.6 Hz, 1H), 4.26 (d, *J* = 5.2 Hz, 1H), 3.70–3.60 (m, 3H), 3.37 (s, 3H), 1.90–1.83 (m, 1H), 1.78–1.63 (m, 4H), 1.16 (d, *J* = 7.0 Hz, 3H); ^13^C­{^1^H} NMR (101 MHz, CDCl_3_, 26 °C): δ 156.7, 136.6, 128.5, 128.0, 127.8, 95.0, 74.3, 67.4, 64.4, 55.5, 51.4, 49.2, 23.5, 20.7, 14.5; IR: ν_max_ (neat, ATR) 3447, 2982, 2946, 2887, 1688, 1670, 1410, 1316, 1297, 1146, 1105, 1073, 1037, 1022, 991 cm^–1^; [α_D_]^25^ = +28.25 (*c* = 0.4, CHCl_3_); HRMS (ESI + qTOF) *m*/*z*: [M + Na]^+^ calcd for C_17_H_25_NNaO_5_^+^ 346.1625; found, 346.1617.

### Procedure for the Oxidation with IBX and Wittig Reaction (**20** and **22**)

To a 5 mL round-bottom flask containing the alcohol **19** (30 mg, 0.093 mmol, 1 equiv) were added ethyl acetate (4 mL, 0.02 M) and 2-iodoxybenzoic acid (IBX) (78 mg, 0.279 mmol, 3 equiv). A reflux condenser was attached, and the reaction mixture was refluxed with vigorous stirring for 5 h. After cooling to room temperature, the reaction mixture was filtered over a small plug of Celite and concentrated under vacuum to furnish the desired aldehyde in quantitative yield. The aldehyde was carefully used in the next step to avoid degradation.

To a 5 mL round-bottom flask containing triphenyl­(undec-10-en-1-yl)­phosphonium bromide **21** (53.5 mg, 0.108 mmol, 1.2 equiv) under an argon atmosphere was added anhydrous THF (1 mL, 0.11 M). The solution was cooled to 0 °C and KHMDS was added (0.1 mmol, 0.2 mL, 0.5 M in toluene, 1.1 equiv). The mixture was stirred at 0 °C for 15 min and more 15 min at room temperature. Then, the mixture was re-cooled to 0 °C and a solution of aldehyde **20** (29 mg, 0.09 mmol, 1 equiv) in anhydrous THF (1 mL, 0.09 M) was added. The reaction mixture was stirred at 0 °C for 1 h and then quenched with saturated aqueous NH_4_Cl solution. The layers were separated, and the aqueous phase was extracted with ethyl acetate (3 × 10 mL). The combined organic phases were dried over anhydrous Na_2_SO_4_, filtered, and concentrated under vacuum. The crude product was purified by column chromatography (silica gel, *n*-hexane:ethyl acetate, 4:1) to furnish **22** as a colorless oil: 50% yield (21 mg), *R*_*f*_ = 0.47 (*n*-hexane:ethyl acetate, 4:1); ^1^H NMR (400 MHz, CDCl_3_, 26 °C): δ 7.38–7.27 (m, 5H), 5.81 (ddt, *J* = 16.9, 10.2, 6.7 Hz, 1H), 5.67 (dd, *J* = 10.9, 9.5 Hz, 1H), 5.38 (dt, *J* = 11.1, 7.7 Hz, 1H), 5.16 (d, *J* = 12.4 Hz, 1H), 5.09 (d, *J* = 12.4 Hz, 1H), 5.02–4.95 (m, 2H), 4.95–4.90 (m, 1H), 4.67 (s, 2H), 4.63–4.51 (m, 1H), 3.68 (dt, *J* = 11.1, 5.1 Hz, 1H), 3.37 (s, 3H), 2.09–1.96 (m, 4H), 1.83–1.63 (m, 4H), 1.36 (dd, *J* = 13.8, 6.3 Hz, 2H), 1.32–1.18 (m, 13H); ^13^C­{^1^H} NMR (101 MHz, CDCl_3_, 26 °C): δ 155.6, 139.2, 136.8, 132.1, 129.7, 128.4, 127.9, 127.8, 114.1, 94.9, 74.4, 67.2, 55.5 (2x), 49.6, 47.1, 33.8, 29.6, 29.5 (2x), 29.3, 29.1, 28.9, 27.3, 21.1, 14.9; IR: ν_max_ (neat, ATR) 2925, 2854, 1696, 1405, 1323, 1296, 1146, 1107, 1067, 1043, 1020, 914 cm^–1^; [α_D_]^25^ = −26.5 (*c* = 0.4, CHCl_3_) HRMS (ESI + qTOF) *m*/*z*: [M + H]^+^ calcd for C_28_H_44_NO_4_^+^ 458.3265; found, 458.3261.

### Procedure for the Wacker Oxidation (**23**)

To a solution of PdCl_2_ (0.46 mg, 2.6 μmol, 0.1 equiv) and CuCl_2_ (2.15 mg, 0.016 mmol, 0.62 equiv) in DMF/water (1:1, 1 mL) was added **22** (12 mg, 0.026 mmol, 1 equiv) in DMF (0.5 mL). Water was added (0.5 mL) and the reaction mixture was stirred under an oxygen atmosphere at room temperature for 17 h. Then, water (2 mL) and diethyl ether (10 mL) were added, the layers were separated, and the aqueous phase was extracted with diethyl ether (5 × 6 mL). The combined organic layers were washed with distilled water (5 × 3 mL) to remove traces of DMF, dried over anhydrous Na_2_SO_4_, filtered, and concentrated under vacuum. The crude product was purified by column chromatography (silica gel, *n*-hexane:ethyl acetate, 7:3) to furnish **23** as a colorless oil: 41% yield (5 mg), *R*_*f*_ = 0.21 (*n*-hexane:ethyl acetate, 4:1); ^1^H NMR (400 MHz, CDCl_3_, 26 °C): δ 7.38–7.28 (m, 5H), 5.67 (dd, *J* = 10.9, 9.5 Hz, 1H), 5.43–5.34 (m, 1H), 5.16 (d, *J* = 12.4 Hz, 1H), 5.09 (d, *J* = 12.4 Hz, 1H), 4.98 (d, *J* = 5.4 Hz, 1H), 4.67 (s, 2H), 4.61–4.53 (m, 1H), 3.68 (dt, *J* = 10.9, 5.3 Hz, 1H), 3.37 (s, 3H), 2.41 (t, *J* = 7.5 Hz, 2H), 2.13 (s, 3H), 1.84–1.62 (m, 4H), 1.31–1.19 (m, 15H, superimposed with “grease”); ^13^C­{^1^H} NMR (101 MHz, CDCl_3_, 26 °C): δ 209.4, 155.5, 136.8, 132.0, 129.7, 128.4, 128.4, 127.9, 127.8, 94.9, 74.4, 67.2, 55.5, 49.6, 47.1, 43.8, 29.9, 29.7, 29.6, 29.5, 29.4, 29.3, 29.2, 27.2, 23.8, 21.0, 14.9; IR: ν_max_ (neat, ATR) 2926, 2853, 1715, 1697, 1463, 1412, 1324, 1298, 1146, 1108, 1069, 1044; [α_D_]^25^ = −35 (*c* = 0.1, CHCl_3_); HRMS (ESI + qTOF) *m*/*z*: [M + Na]^+^ calcd for C_28_H_43_NNaO_5_^+^ 496.3033; found, 496.3033.

### Procedure for the Double Bond Reduction and Deprotection (**26**)

To a 5 mL round-bottom flask containing Pd/C (0.67 mg, 0.63 μmol, 0.1 equiv, 10 wt % on carbon) was added a solution of **23** (3 mg, 6.3 μmol, 1 equiv) in MeOH (3 mL, 2 mM). The reaction mixture was stirred under a hydrogen atmosphere at room temperature for 16 h. Then, the reaction flask was opened and a few drops of pure HCl were added. After 24 h of stirring at room temperature, the mixture was filtered over a small cotton plug and the solvent was removed under vacuum. The residue was dissolved in 2 mL of H_2_O, washed with Et_2_O (3 × 2 mL), basified with a 10% NaOH solution to reach pH = 10, and extracted with CH_2_Cl_2_ (3 × 2 mL). The combined organic layers were dried over anhydrous Na_2_SO_4_, filtered, and concentrated to afford (−)-cassine as a colorless oil: 78% yield (1.5 mg). ^1^H NMR (500 MHz, CDCl_3_, 26 °C): δ 3.56 (br s, 1H), 2.77 (q, *J* = 6.1 Hz, 1H), 2.59–2.52 (m, 1H), 2.42 (t, *J* = 7.5 Hz, 2H), 2.13 (s, 3H), 1.93–1.88 (m, 1H), 1.64–1.45 (m, 5H + water), 1.33–1.24 (m, 16H + “grease”), 1.11 (d, *J* = 6.5 Hz, 3H), ^13^C­{^1^H} NMR (126 MHz, CDCl_3_, 26 °C): δ 209.4, 68.0, 57.3, 55.9, 43.8, 36.9, 32.0, 29.9, 29.8, 29.5, 29.5, 29.4 (2*x*), 29.2, 26.0, 25.8, 23.9, 18.6; [α_D_]^25^ = −6.0 (*c* = 0.3, CHCl_3_) (lit:^5e^ −6.5 (*c* = 0.6, CHCl_3_); HRMS (ESI + qTOF) *m*/*z*: [M + H]^+^ calcd for C_18_H_36_NO_2_^+^ 298.2741; found, 298.2752.

## Supplementary Material



## Data Availability

The data underlying this study are available in the published article and its Supporting Information.

## Notes

Tables containing the conditions tested for protection of **12** and oxidation studies of **16**; copies of ^1^H, ^13^C­{^1^H}, and NOESY1D spectra of new and final known products; and theoretical calculations details (PDF)
