# Supplementary material for: α,β-Unsaturated Diazoketones as Building Blocks to Piperidine Alkaloids: Total Synthesis of (−)-Cassine
Source: J Org Chem. 2025 Jun 10;90(24):8420–8. doi: 10.1021/acs.joc.5c00729 (PMC12186531; doi:10.1021/acs.joc.5c00729)
Supplement: Supplementary file 1 [file jo5c00729_si_001.pdf]

Supporting Information

α,β-Unsaturated Diazoketones as Building Blocks to Piperidine Alkaloids: Total Synthesis of (-)-Cassine

João Pedro de F. Lima, Rafael D. C. Gallo, Antonio C. B. Burtoloso*

Institute of Chemistry of São Carlos, University of São Paulo, CEP 13563-120, São Carlos, SP, Brazil

e-mail: Antonio C. B. Burtoloso: antonio@iqsc.usp.br

Table of Contents

Cautionary Note…………………………………………………………………S3

Table S1: Conditions for protection of allylic alcohol **12**…………………………...S3

Table S2: Oxidation studies of **16**……………………………………………….…...S4

^{1}H and ^{13}C{^{1}H} spectra of compound (**1**)...S5

^{1}H and ^{13}C{^{1}H} spectra of compound (**3**)...S6

^{1}H and ^{13}C{^{1}H} spectra of compound (**4**)...S7

^{1}H and ^{13}C{^{1}H} spectra of compound (**6**)...S8

^{1}H and ^{13}C{^{1}H} spectra of compound (**7**)...S9

^{1}H and ^{13}C{^{1}H} spectra of compound (**8**)..S10

^{1}H and ^{13}C{^{1}H} spectra of compound (**9**)..S11

^{1}H and ^{13}C{^{1}H} spectra of compound (**10**)...S12

^{1}H and ^{13}C{^{1}H} spectra of compound (**11**)...S13

^{1}H and ^{13}C{^{1}H} spectra of compound (**12**)...S14

^{1}H and ^{13}C{^{1}H} spectra of compound (**15**)...S15

^{1}H and ^{13}C{^{1}H} spectra of compound (**16**)..S16

^{1}H and ^{13}C{^{1}H} spectra of compound (**19**)..S17

^{1}H and ^{13}C{^{1}H} spectra of compound (**22**)..S18

^{1}H and ^{13}C{^{1}H} spectra of compound (**23**)..S19

^{1}H and ^{13}C{^{1}H} spectra of (-)-Cassine (**26**)..S20

Table S3: ^{1}H NMR data vs literature…………………………….…….……S21

Table S4: ^{13}C{^{1}H} NMR data vs literature……………………………..……....S22

NOE studies of compound (**9**)...S23

NOE studies of compound (**12**)………………………………………….……......S24

Calculations..……………………...…………………………………………... …S25

Cautionary Note:

o-Iodoxybenzoic acid (IBX) is an oxidizer and may pose a risk of explosion under certain conditions, especially when dry or finely powdered. It should be handled in small quantities and stored in a tightly sealed container away from heat or friction.

n-Butyllithium (n-BuLi) is a pyrophoric reagent, reacts violently with water and must be handled under an inert atmosphere using air-free techniques.

Hydrogen is classified as a GHS Flammable Gas, Category 1. Hydrogen balloon was handled in a fume hood with exhaust and away from flammable materials.

Diazophosphonates **1**, **2**, **3** and **4** can be stored in a refrigerator at 4 °C for several months without degradation and did not exhibit explosive behavior during the course of this work.

Table S1: Protection of the allylic alcohol **12**.

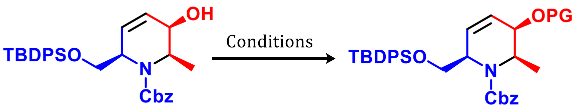

(12) PG = Bn (13)
p-NO_2-Bz (14)
MOM (15)

Entry	Conditions	Yield
1	BnBr (1.5 eq.), NaH (1.1 eq.), THF, 0 °C to reflux, 24h	-
2	BnBr (2 eq.), NaH (1.5 eq.), DMF, 0 °C to 25 °C, 16h	-
3	BnBr (2 eq.), NaH (1.5 eq.), TBAI (0.1 eq.), DMF, 0 °C to 25 °C, 16h	29%
4	BnBr (2 eq.), NaH (5 eq.), DMF, 0 °C to 25 °C, 7h	-
5	BnBr (1.5 eq.), KHMDS (1.5 eq.), THF, 0 °C to reflux, 24h	21%
6	BnBr (1.5 eq.), Ag_2O (2 eq.), KI (0.1 eq.), Et_2O, 25 °C, 16h	-
7	BnBr (1.5 eq.), Ag_2O (2 eq.), TBAI (0.1 eq.), DMF, 25 °C, 16h	-
8	$BnOCNHCl_3$ (1.5 eq.), $Sc(OTf)_3$ (0.05 eq.), MePh, 25 °C, 16h	-
9	$BnOCNHCl_3$ (1.5 eq.), TfOH (0.05 eq.), Et_2O, 25 °C, 16h	-
10	BnOCOCl (1.6 eq.), DMAP (0.13 eq.), Et_3N (5 eq.), THF, 25 °C, 16h	-
11	BnOCOCl (2.1 eq.), TMEDA (0.6 eq.), CH_2Cl_2, 0 °C – 25 °C, 16h	-
12	*p*-NO_2BzCl (1.6 eq.), DMAP (0.13 eq.), Py (5 eq.), CH_2Cl_2, 25 °C, 16h	22%
13	*p*-NO_2BzCl (1.3 eq.), DMAP (0.13 eq.), Et_3N (5 eq.), CH_2Cl_2, 25 °C, 5h	65%
14	MOMCl (5 eq.), DIPEA (5 eq.), CH_2Cl_2, reflux, 24h	53%
15	MOMCl (5 eq.), DIPEA (5 eq.), TBAI (0.1 eq.), CH_2Cl_2, reflux, 24h	79%

Table S2: Oxidation studies of **16**.

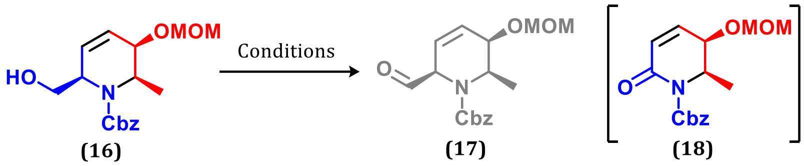

Entry	Conditions	Yield
1	IBX (3 equiv), AcOEt, reflux, 5h	Complex mixture
2	Dess-Martin Periodinane (1.5 equiv), DCM, H_2O (cat.), 25 ºC, 16h	Complex mixture
3	DMSO (2.5 equiv), $(COCl)_2$ (1.1 equiv), Et_3N (4 equiv), DCM, -72 ºC to 25 ºC, 5h	-
4	SO_3Py (4 equiv), Et_3N (10 equiv), DCM/DMSO (5:1), 0 ºC to 25 ºC, 24h	-
5	TPAP (0.1 equiv), NMO (1.5 equiv), molecular sieves (500 mg/mmol), ACN, 25 ºC, 16h	-
6	TPAP (0.1 equiv), NMO (1.5 equiv), mol. sieves (500 mg/mmol), DCM:ACN (9:1), 2 5ºC, 16h	-
7*	PCC (4 equiv), Celite (1.3 g/mmol), DCM, 0 ºC to 25 ºC, 12h	75%

*Yield for product **18**.

^{1}H (500 MHz, CDCl$_3$) *(3-diazo-2-oxobutyl)phosphonate* **(1)**

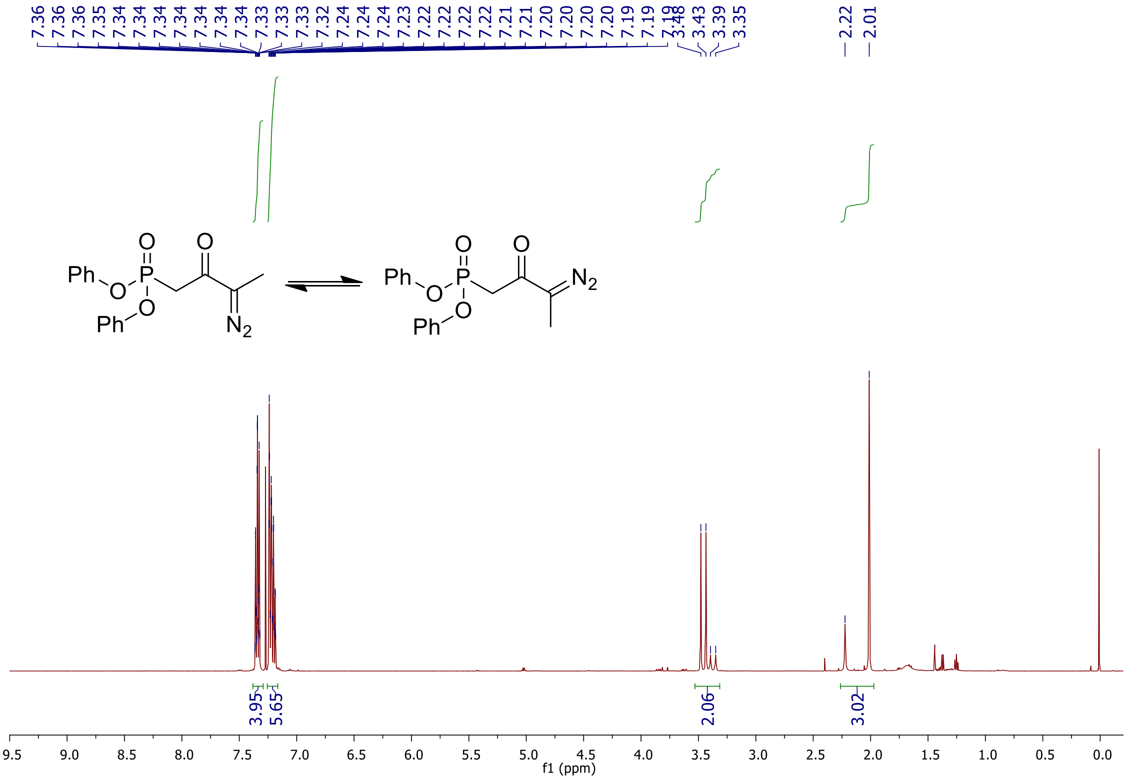

^{13}C{^{1}H} (126 MHz, CDCl$_3$) *(3-diazo-2-oxobutyl)phosphonate* **(1)**

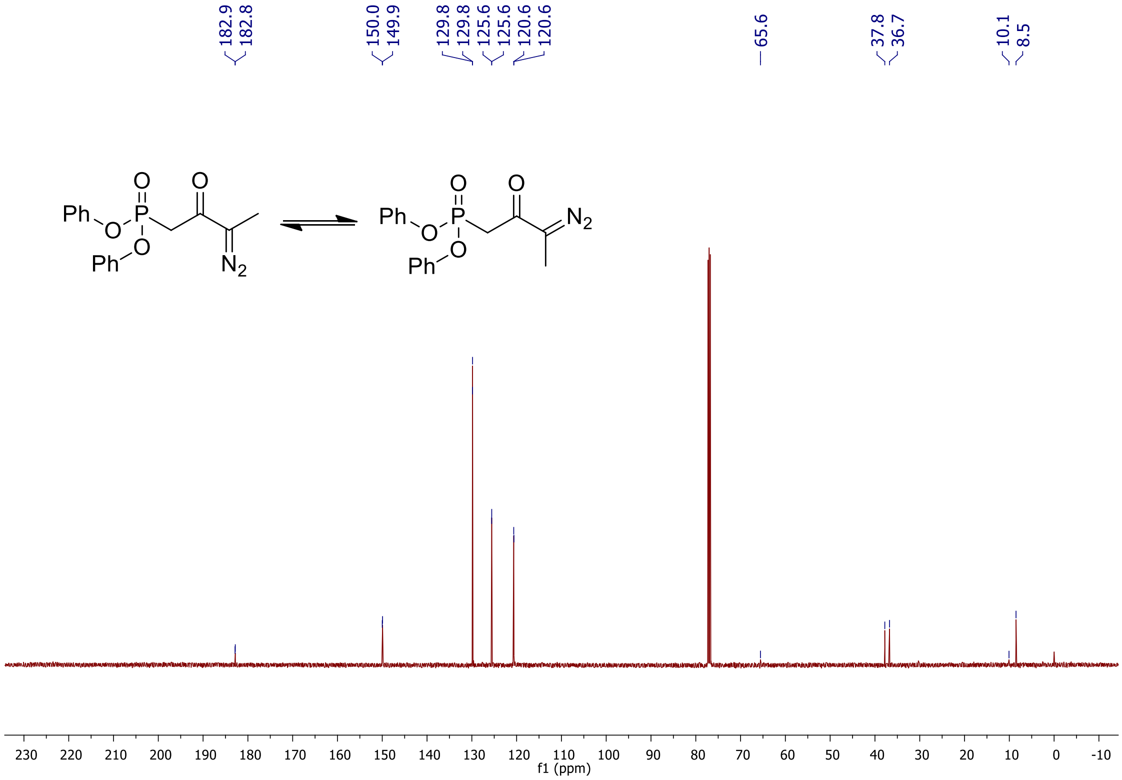

^{1}H (500 MHz, CDCl$_3$) *(3-diazo-2-oxo-4-phenylbutyl)phosphonate* **(3)**

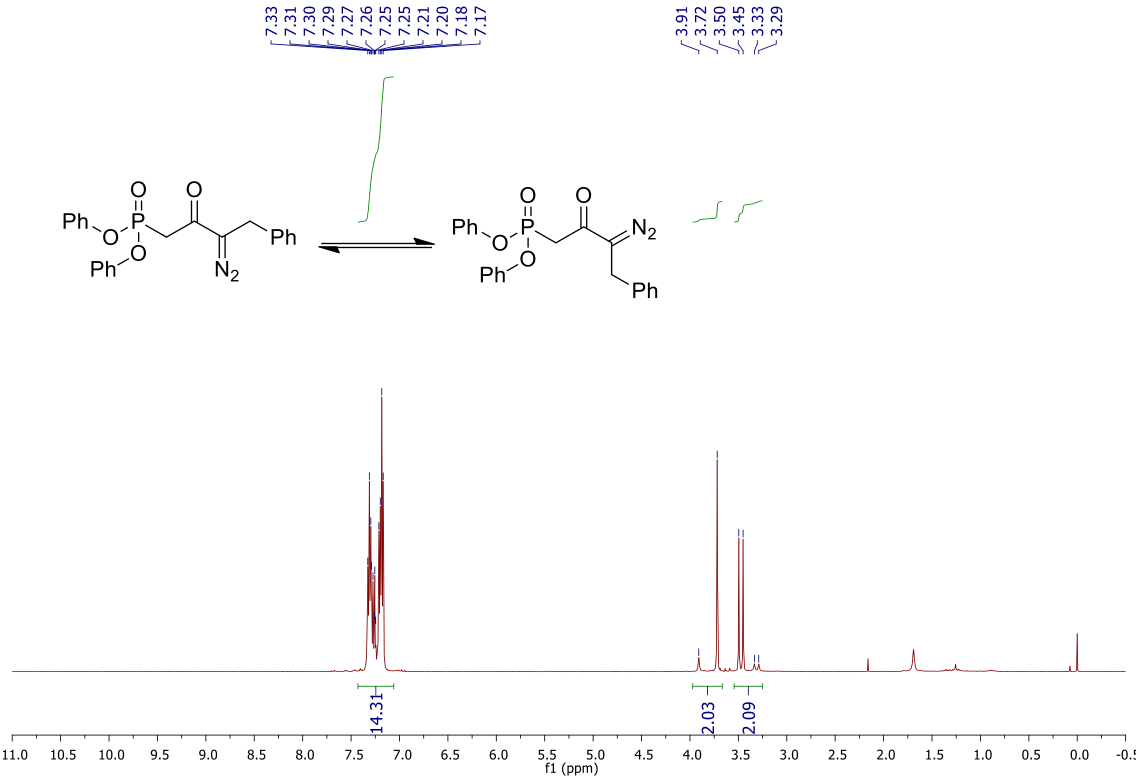

^{13}C{^{1}H} (126 MHz, CDCl$_3$) *(3-diazo-2-oxo-4-phenylbutyl)phosphonate* **(3)**

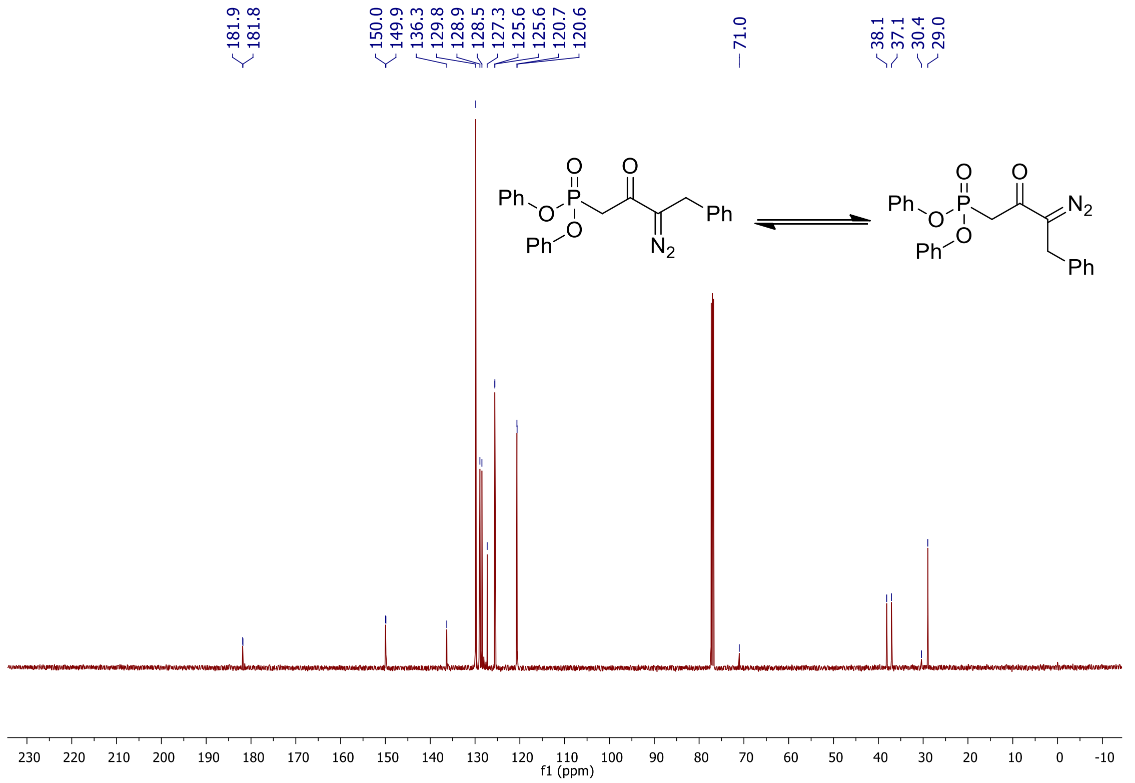

^{1}H (500 MHz, CDCl$_3$) *(3-diazo-2-oxohex-5-en-1-yl)phosphonate* **(4)**

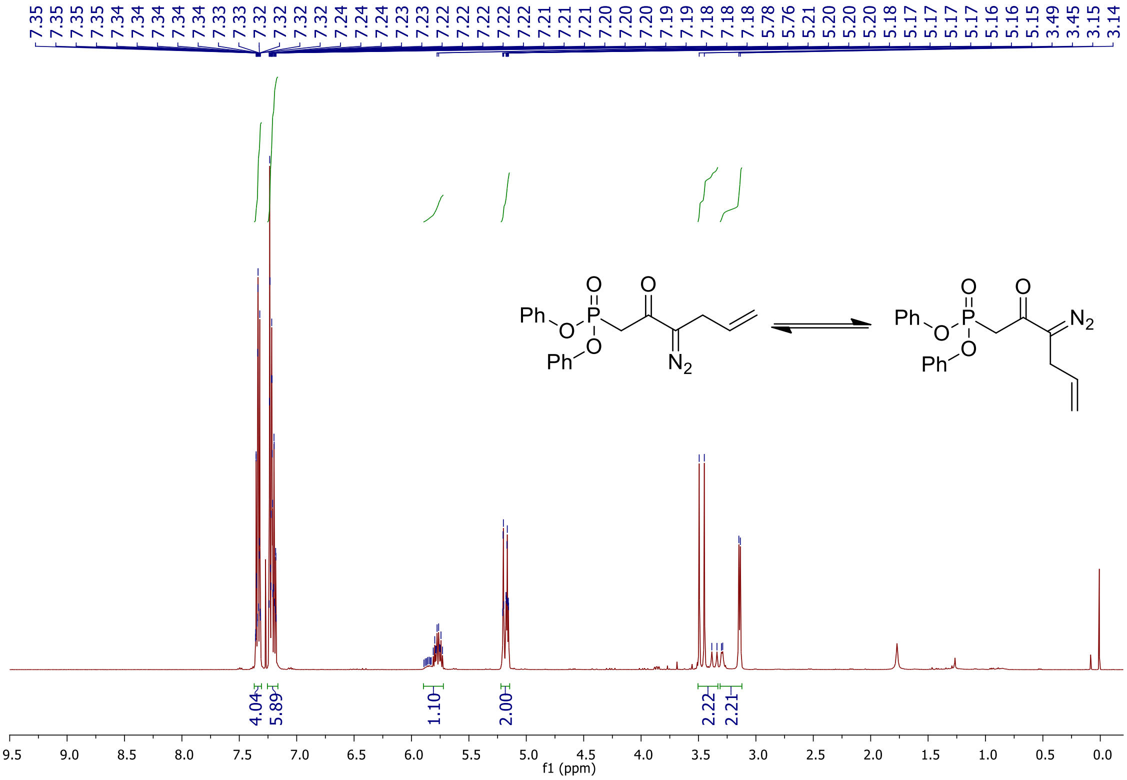

^{13}C{^{1}H} (126 MHz, CDCl$_3$) *(3-diazo-2-oxohex-5-en-1-yl)phosphonate* **(4)**

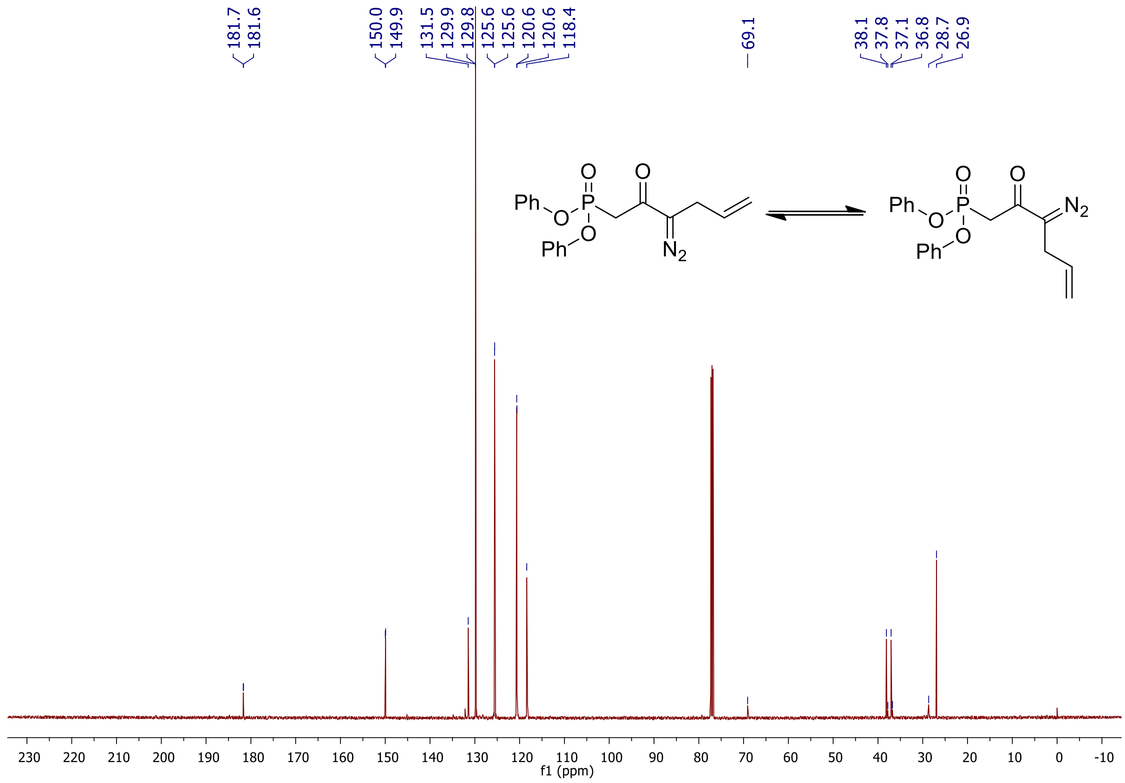

^{1}H (500 MHz, DMSO-*d*$_6$, 80°C) *(R,Z)-benzyl (1-((tert-butyldiphenylsilyl)oxy)-6-diazo-5-oxohept-3-en-2-yl)carbamate* **(6)**

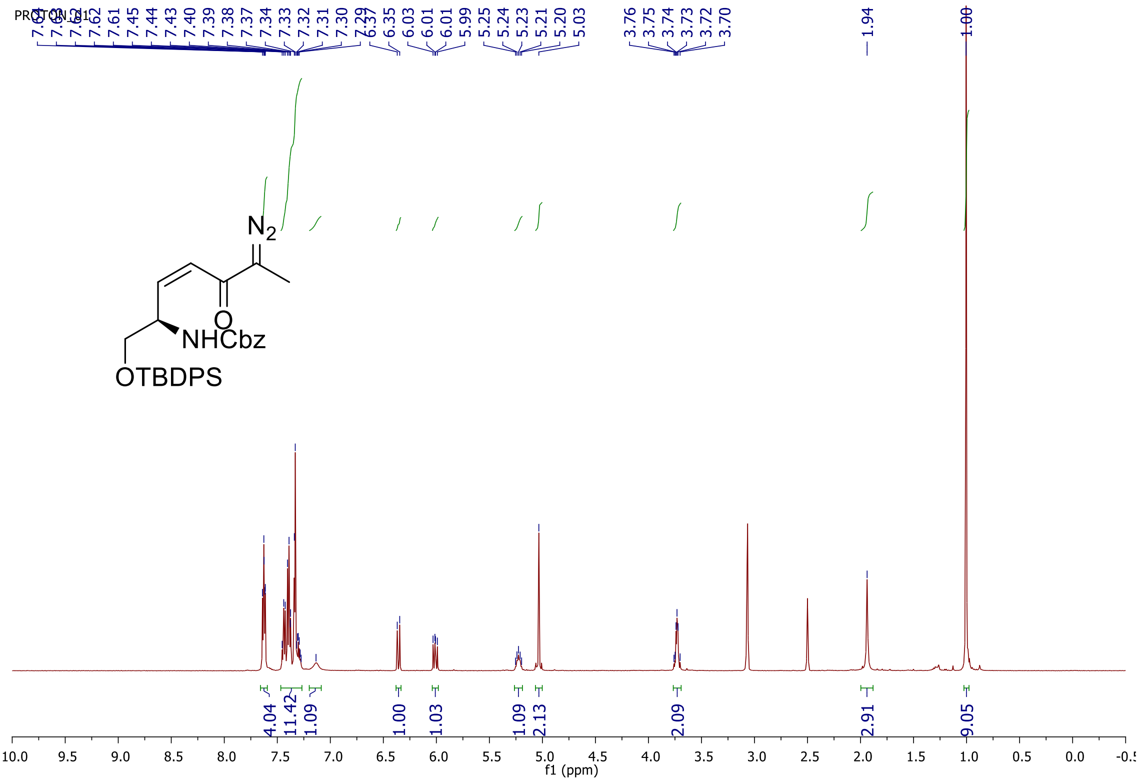

^{13}C{^{1}H} (126 MHz, DMSO-*d*$_6$, 80°C) *(R,Z)-benzyl (1-((tert-butyldiphenylsilyl)oxy)-6-diazo-5-oxohept-3-en-2-yl)carbamate* **(6)**

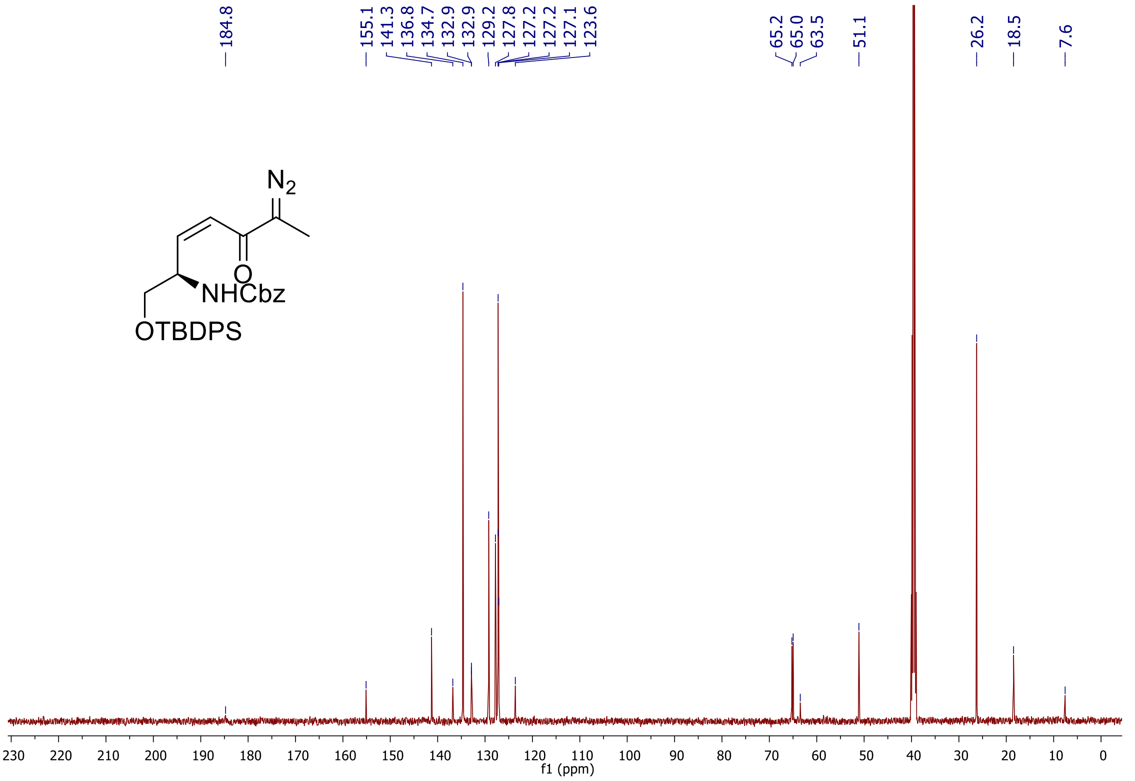

^{1}H (500 MHz, DMSO-*d*$_6$, 80ºC) *(R,Z)-benzyl (1-((tert-butyldiphenylsilyl)oxy)-6-diazo-5-oxo-7-phenylhept-3-en-2-yl)carbamate* **(7)**

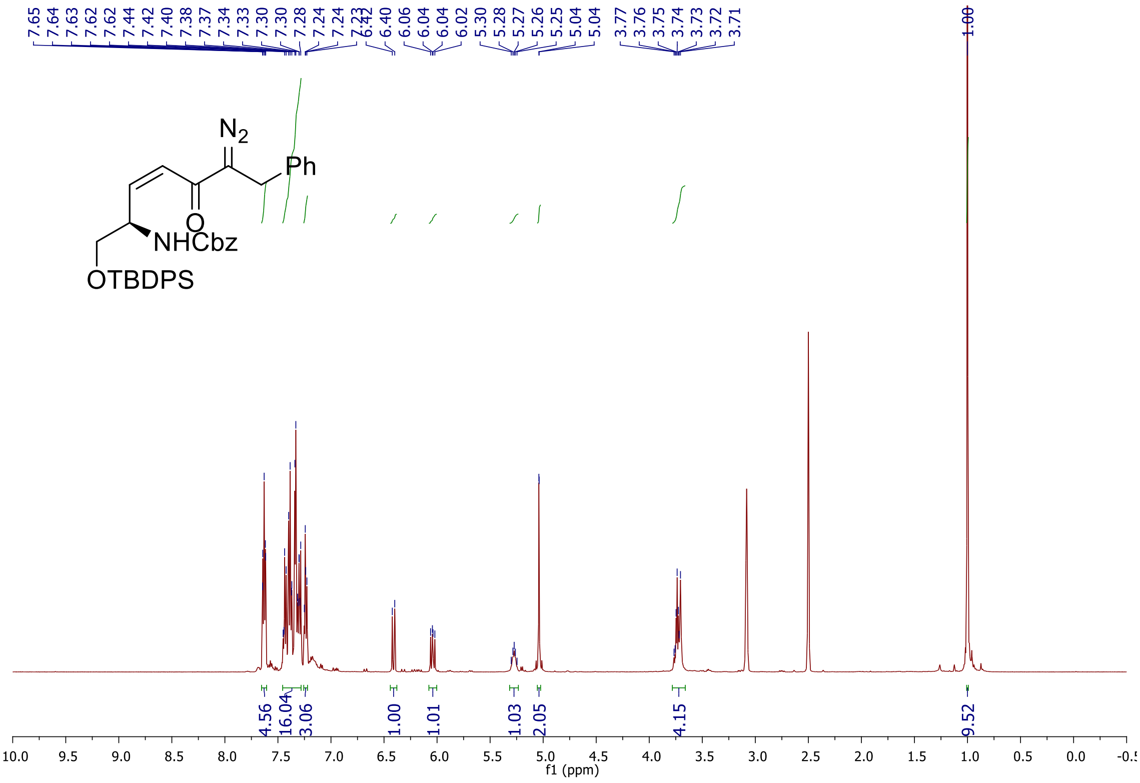

^{13}C{^{1}H} (126 MHz, DMSO-*d*$_6$, 80ºC) *(R,Z)-benzyl (1-((tert-butyldiphenylsilyl)oxy)-6-diazo-5-oxo-7-phenylhept-3-en-2-yl)carbamate* **(7)**

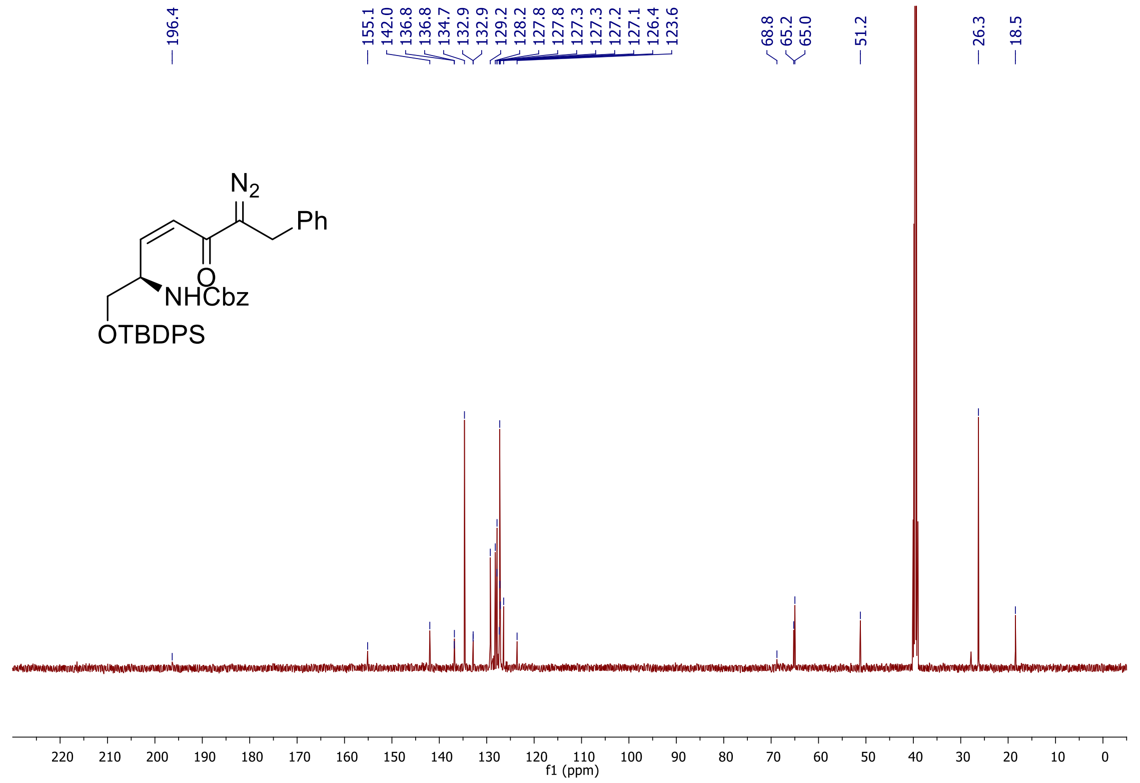

^{1}H (500 MHz, DMSO-*d*$_6$, 80°C) *(R,Z)-benzyl (1-((tert-butyldiphenylsilyl)oxy)-6-diazo-5-oxonona-3,8-dien-2-yl)carbamate* **(8)**

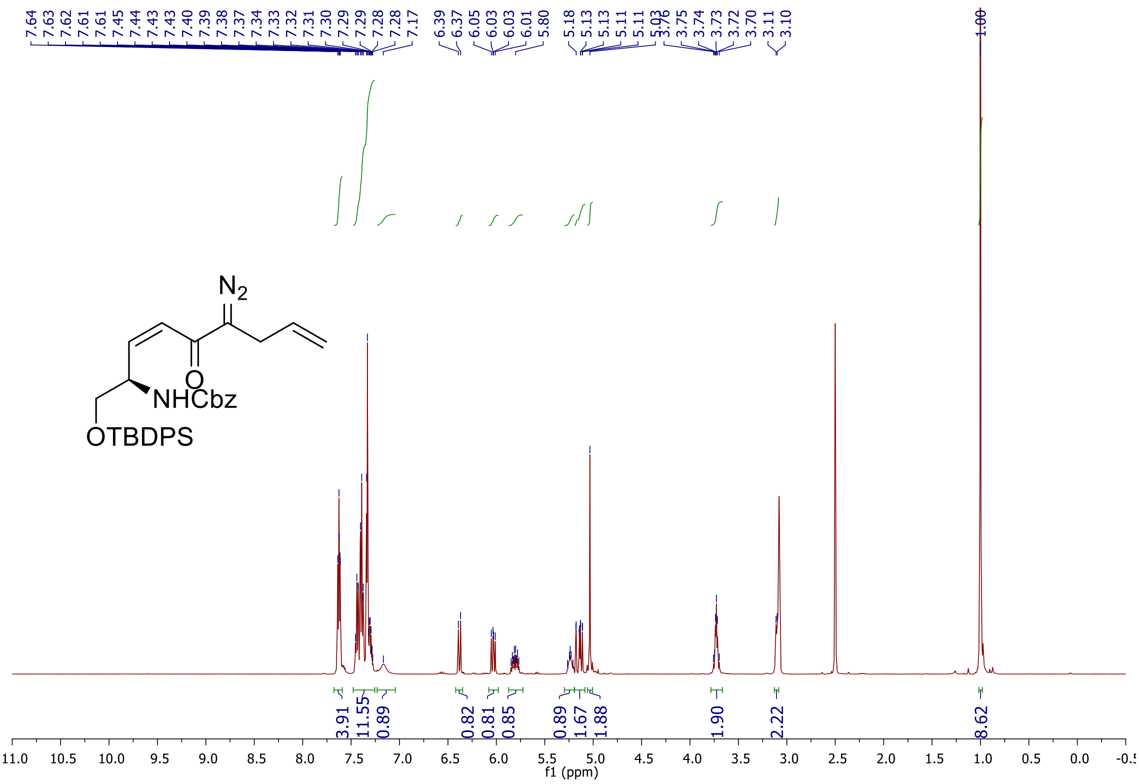

^{13}C{^{1}H} (126 MHz, DMSO-*d*$_6$, 80°C) *(R,Z)-benzyl (1-((tert-butyldiphenylsilyl)oxy)-6-diazo-5-oxonona-3,8-dien-2-yl)carbamate* (**8**)

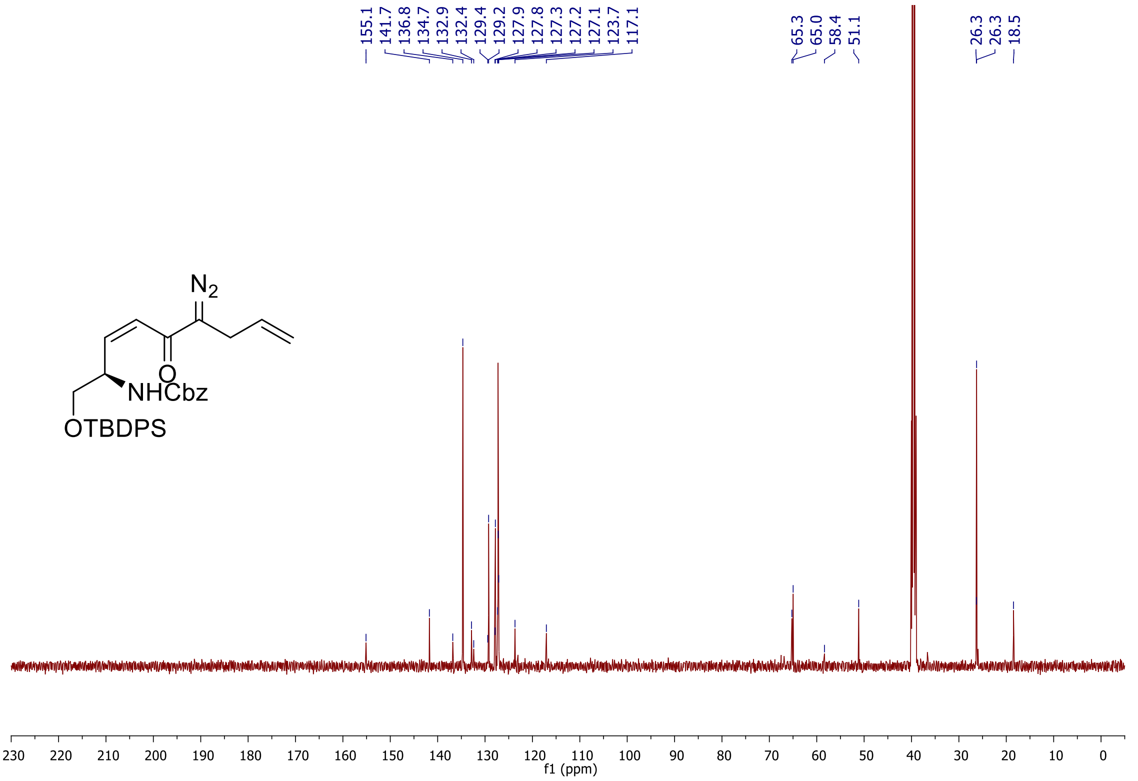

^{1}H (500 MHz, DMSO-*d*$_6$, 80°C) *(2R,6R)-benzyl 2-(((tert-butyldiphenylsilyl)oxy)methyl)-6-methyl-5-oxo-5,6-dihydropyridine-1(2H)-carboxylate* **(9)**

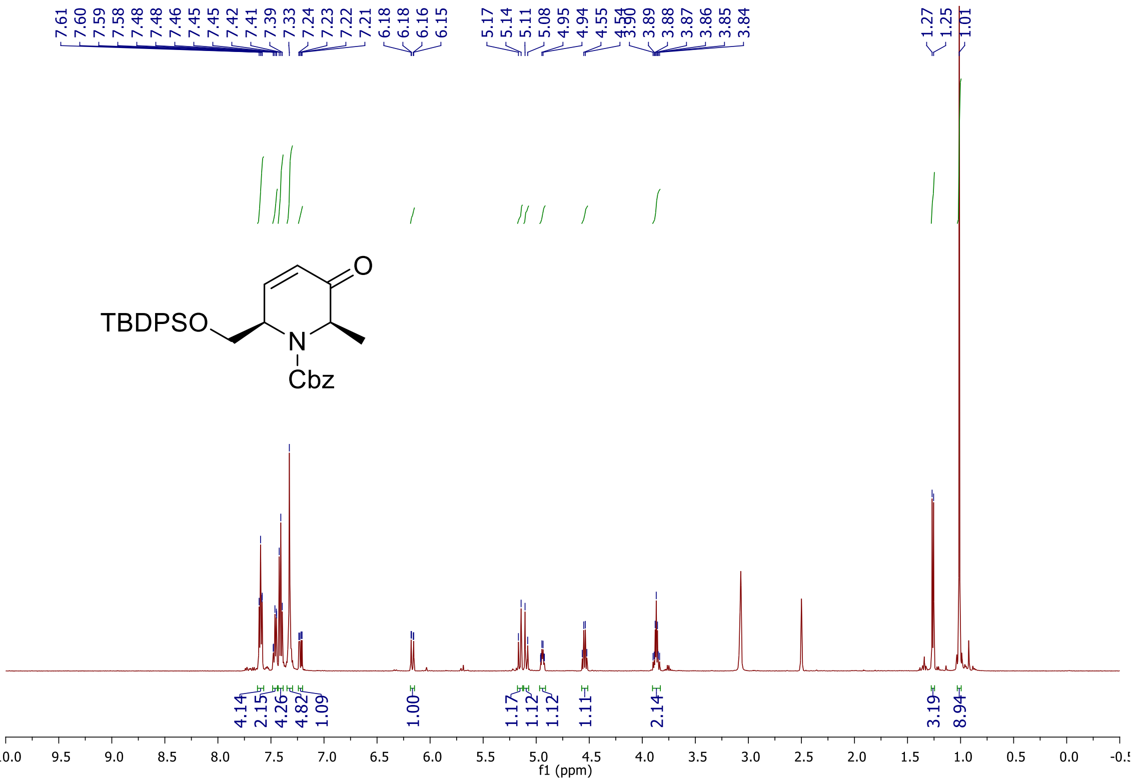

^{13}C{^{1}H} (126 MHz, DMSO-*d*$_6$, 80°C) *(2R,6R)-benzyl 2-(((tert-butyldiphenylsilyl)oxy)methyl)-6-methyl-5-oxo-5,6-dihydropyridine-1(2H)-carboxylate* **(9)**

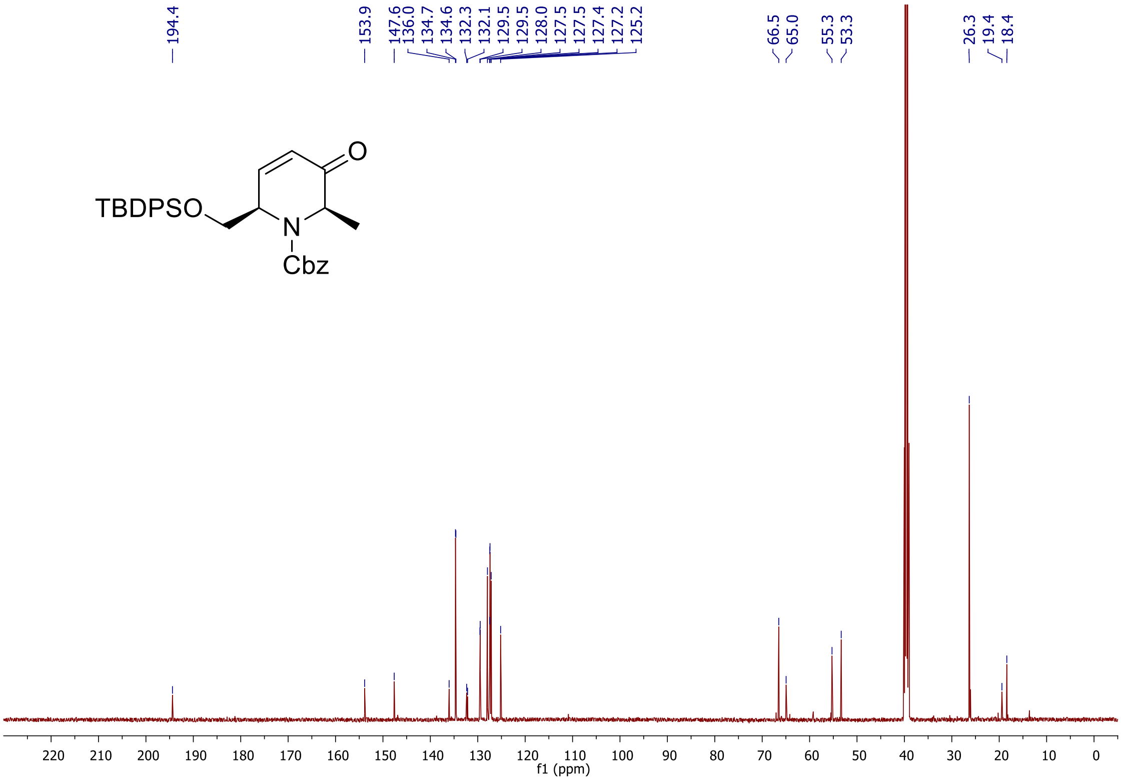

^{1}H (500 MHz, DMSO-*d*$_6$, 80°C) *(2R,6R)-benzyl 6-benzyl-2-(((tert-butyldiphenylsilyl)oxy)methyl)-5-oxo-5,6-dihydropyridine-1(2H)-carboxylate* **(10)**

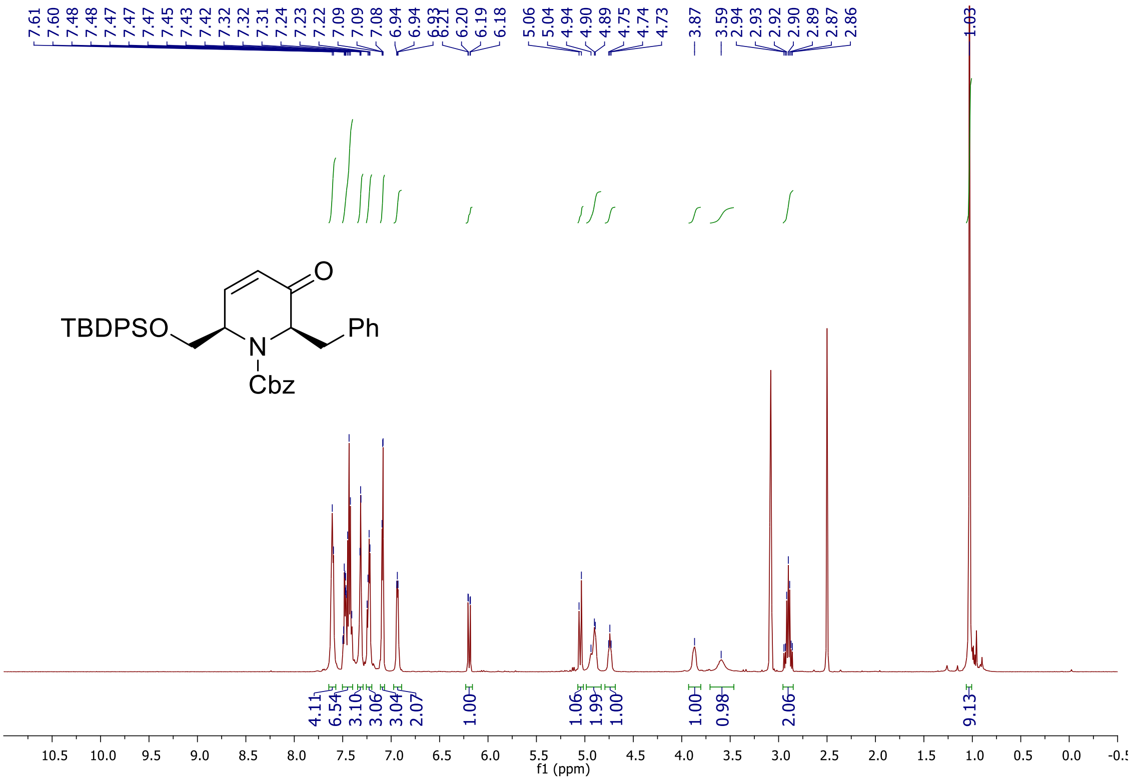

^{13}C{^{1}H} (126 MHz, DMSO-*d*$_6$, 80°C) *(2R,6R)-benzyl 6-benzyl-2-(((tert-butyldiphenylsilyl)oxy)methyl)-5-oxo-5,6-dihydropyridine-1(2H)-carboxylate* **(10)**

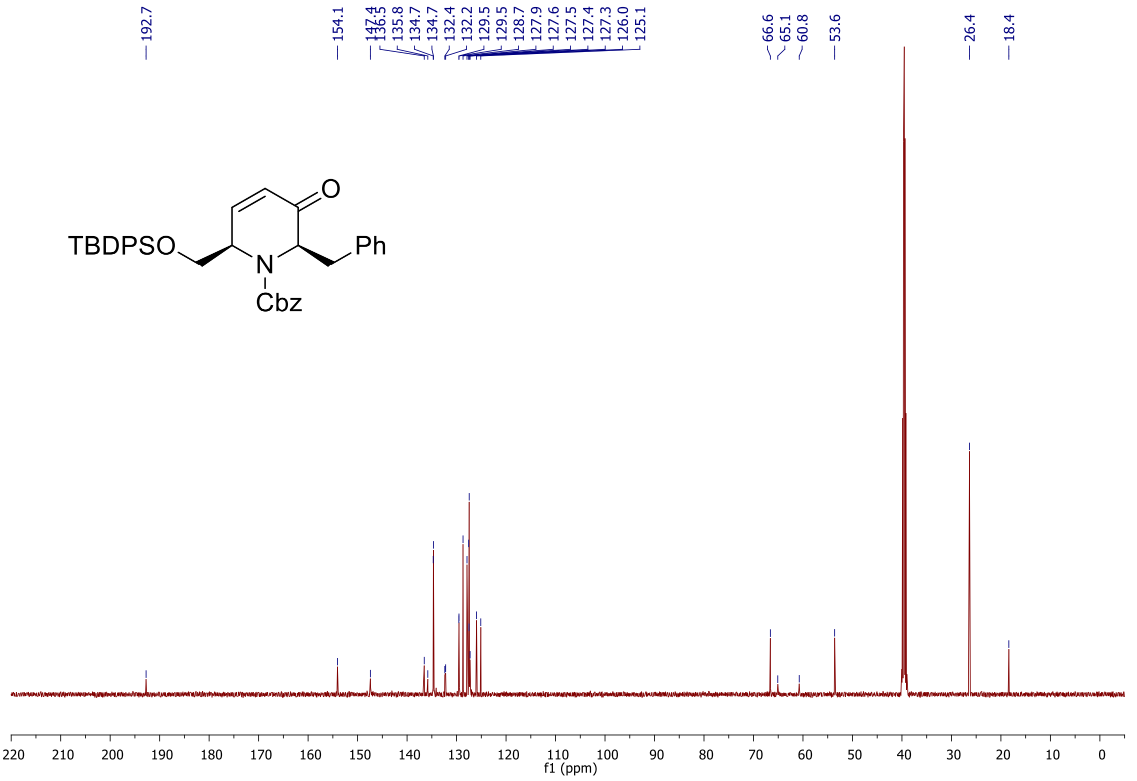

^{1}H (500 MHz, DMSO-d_6, 80°C) *(2R,6R)-benzyl 6-allyl-2-(((tert-butyldiphenylsilyl)oxy)methyl)-5-oxo-5,6-dihydropyridine-1(2H)-carboxylate* **(11)**

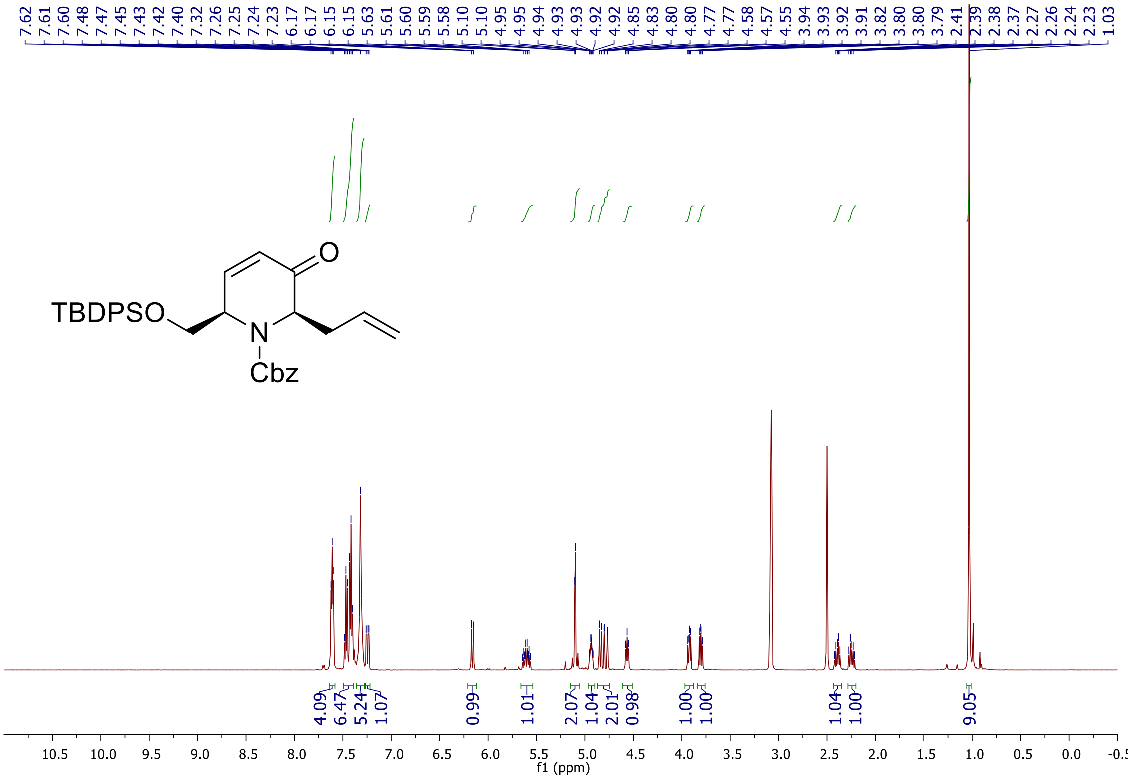

^{13}C{^{1}H} (126 MHz, DMSO-d_6, 80°C) *(2R,6R)-benzyl 6-allyl-2-(((tert-butyldiphenylsilyl)oxy)methyl)-5-oxo-5,6-dihydropyridine-1(2H)-carboxylate* **(11)**

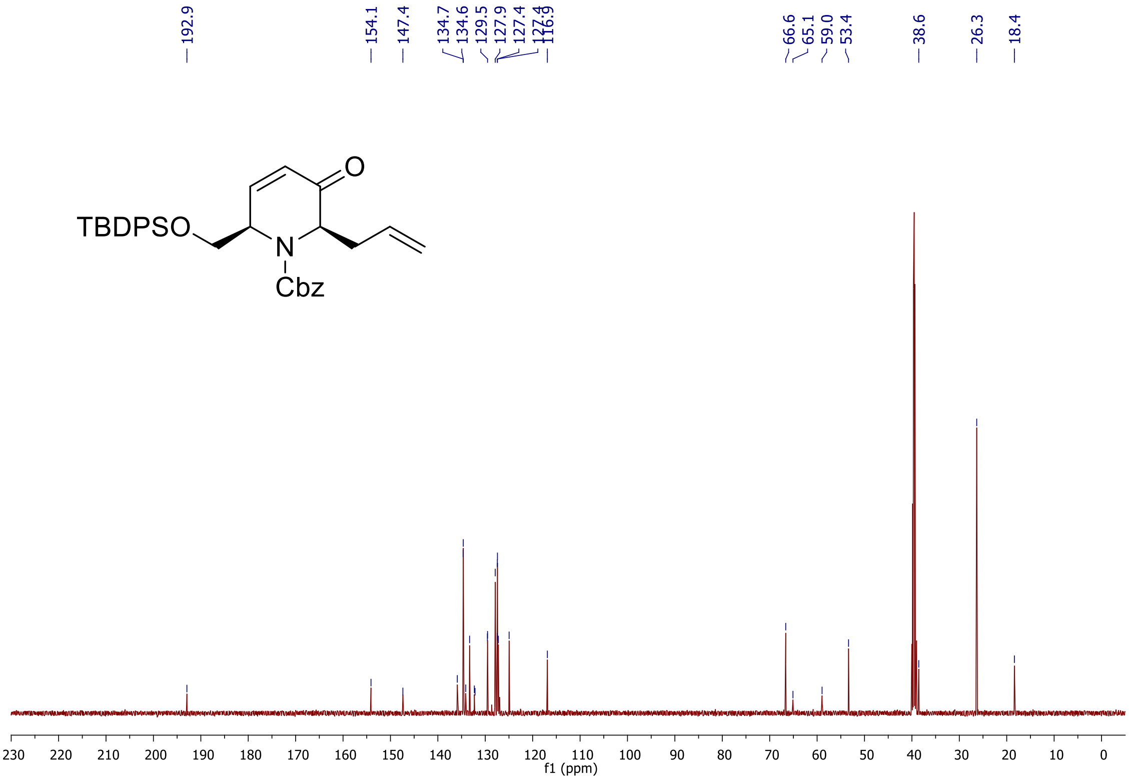

^{1}H (500 MHz, DMSO-*d*$_6$, 80°C) and $^{13}C\{^{1}H\}$ (126 MHz, DMSO-*d*$_6$, 80°C)

(2R,5R,6R)-benzyl 2-(((tert-butyldiphenylsilyl)oxy)methyl)-5-hydroxy-6-methyl-5,6-dihydropyridine-1(2H)-carboxylate **(12)**

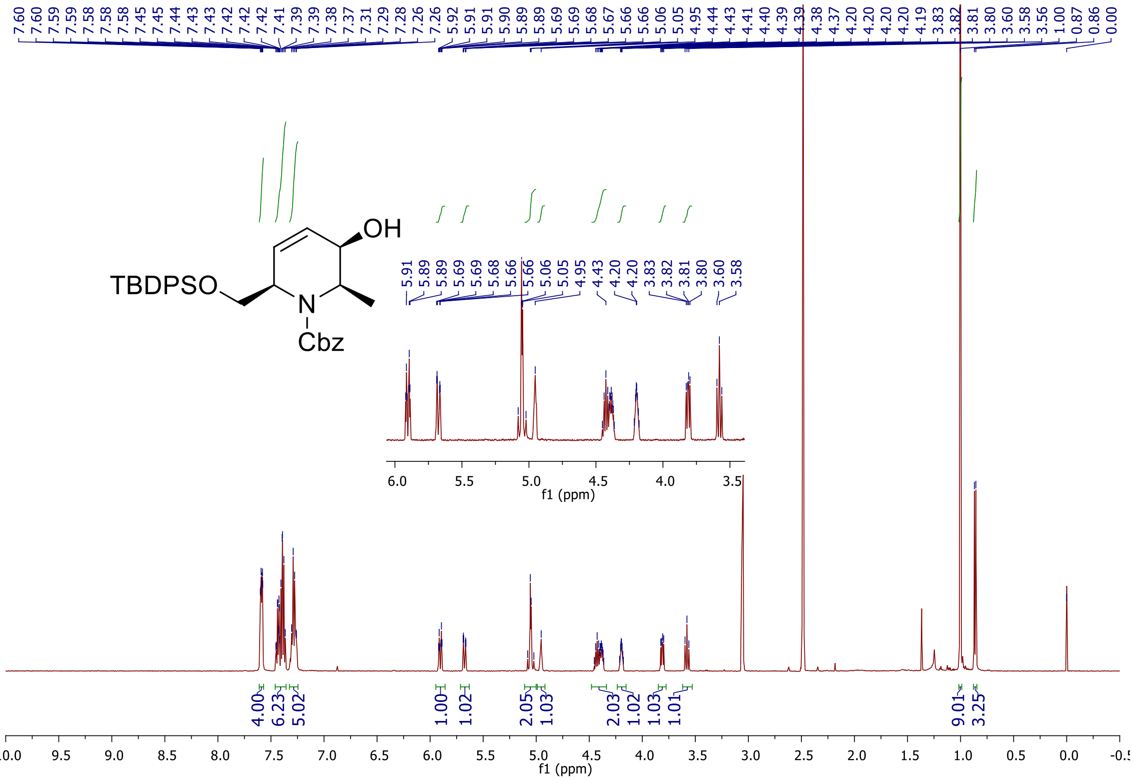

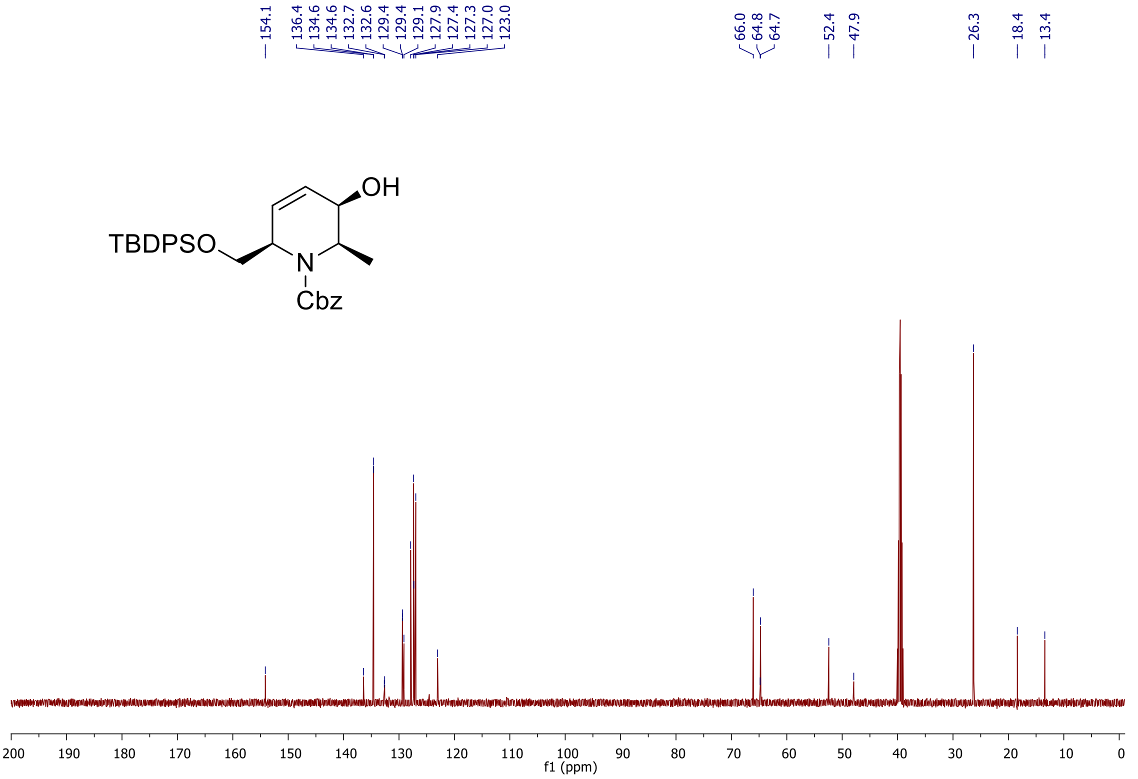

^{1}H (500 MHz, DMSO-*d*$_6$, 80°C) and ^{13}C{^{1}H} (126 MHz, DMSO-*d*$_6$, 80°C)

(2R,5R,6R)-benzyl 2-(((tert-butyldiphenylsilyl)oxy)methyl)-5-(methoxymethoxy)-6-methyl-5,6-dihydropyridine-1(2H)-carboxylate **(15)**

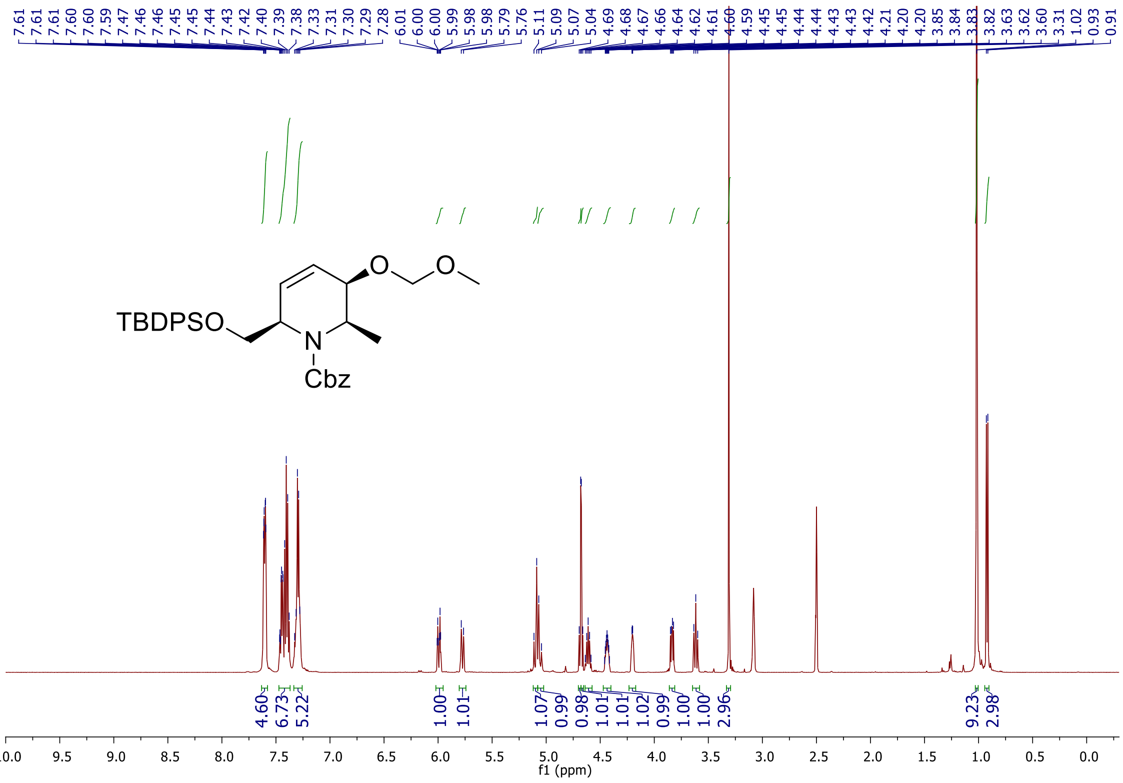

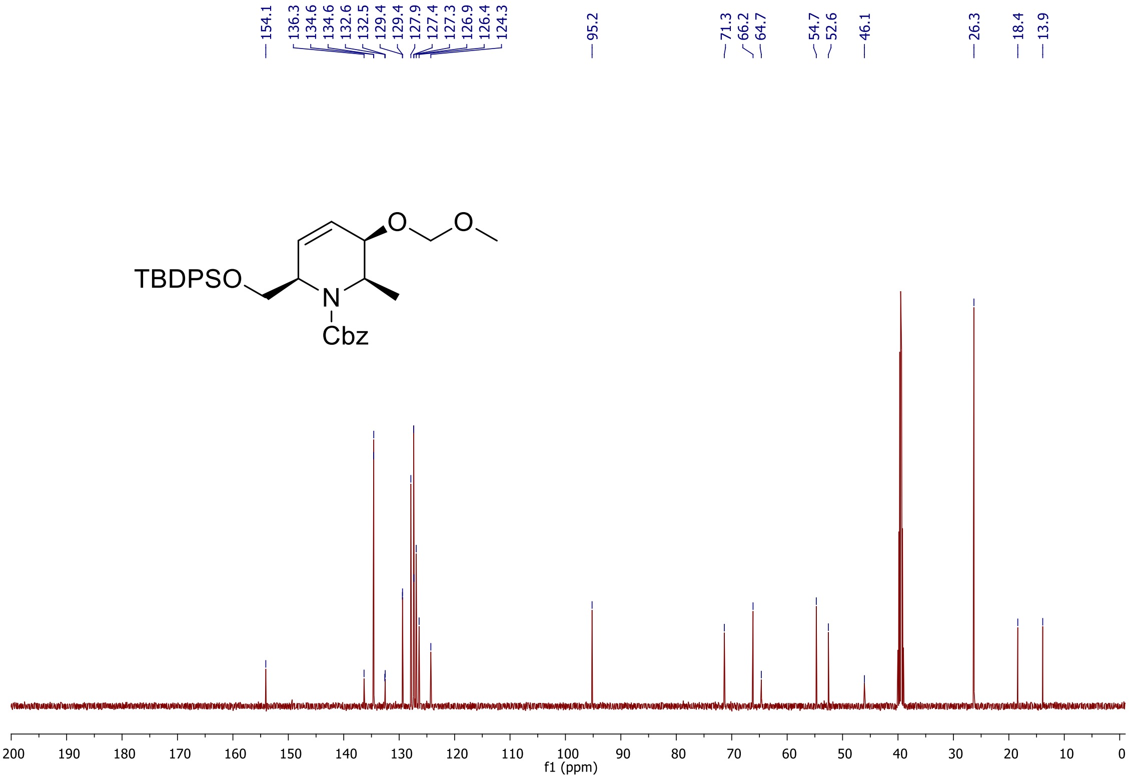

^{1}H (400 MHz, CDCl$_3$, 26ºC) *(2R,5R,6R)-benzyl 2-(hydroxymethyl)-5-(methoxymethoxy)-6-methyl-5,6-dihydropyridine-1(2H)-carboxylate* **(16)**

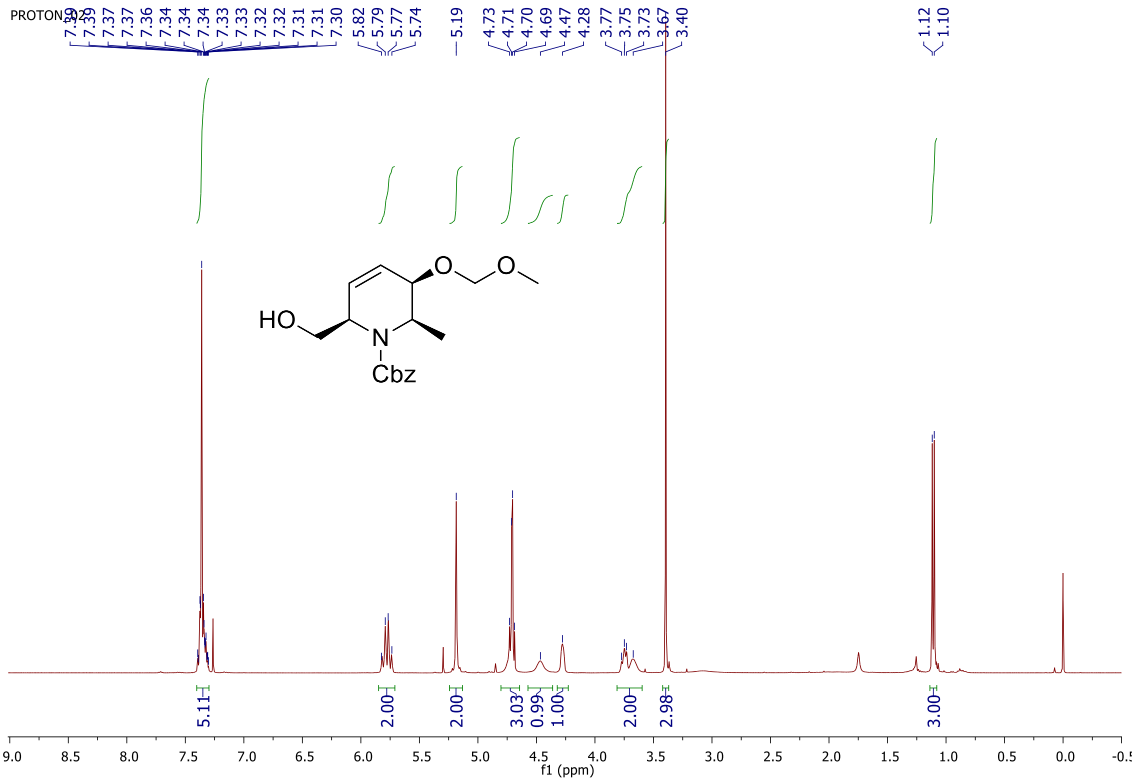

^{13}C{^{1}H} (101 MHz, CDCl$_3$, 26ºC) *(2R,5R,6R)-benzyl 2-(hydroxymethyl)-5-(methoxymethoxy)-6-methyl-5,6-dihydropyridine-1(2H)-carboxylate* **(16)**

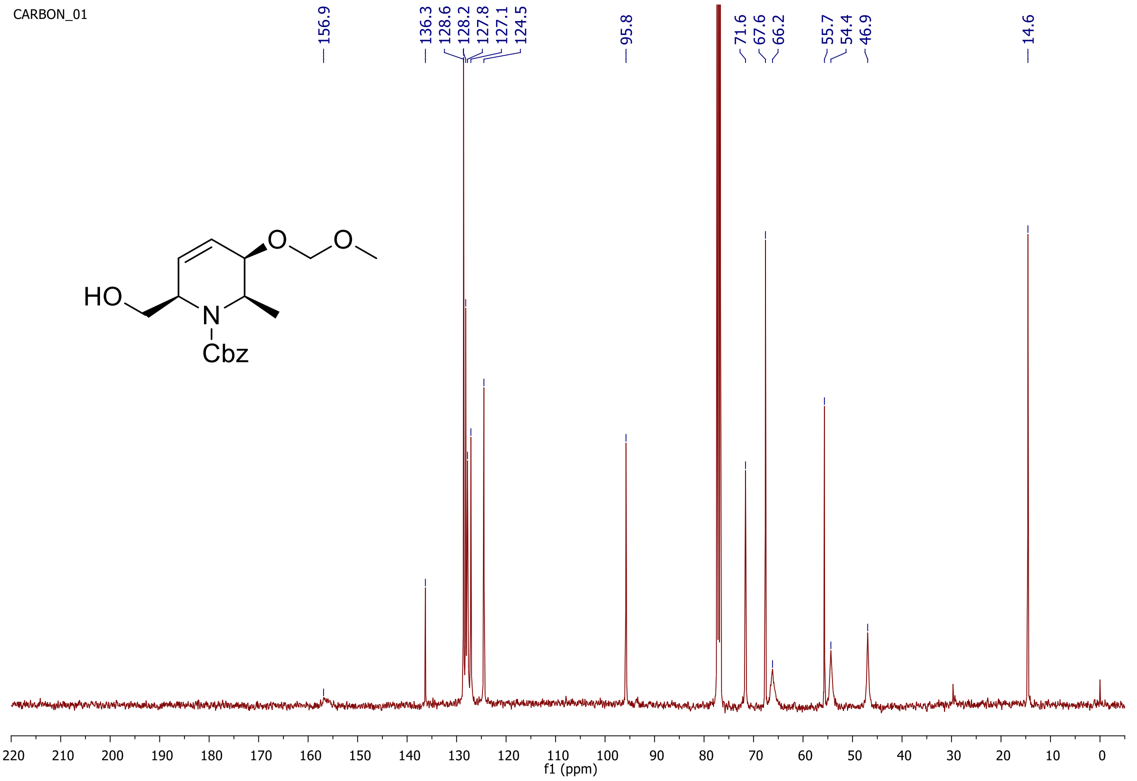

^{1}H (400 MHz, CDCl$_3$, 26ºC) *(2R,3R,6R)-benzyl 6-(hydroxymethyl)-3-(methoxymethoxy)-2-methylpiperidine-1-carboxylate* **(19)**

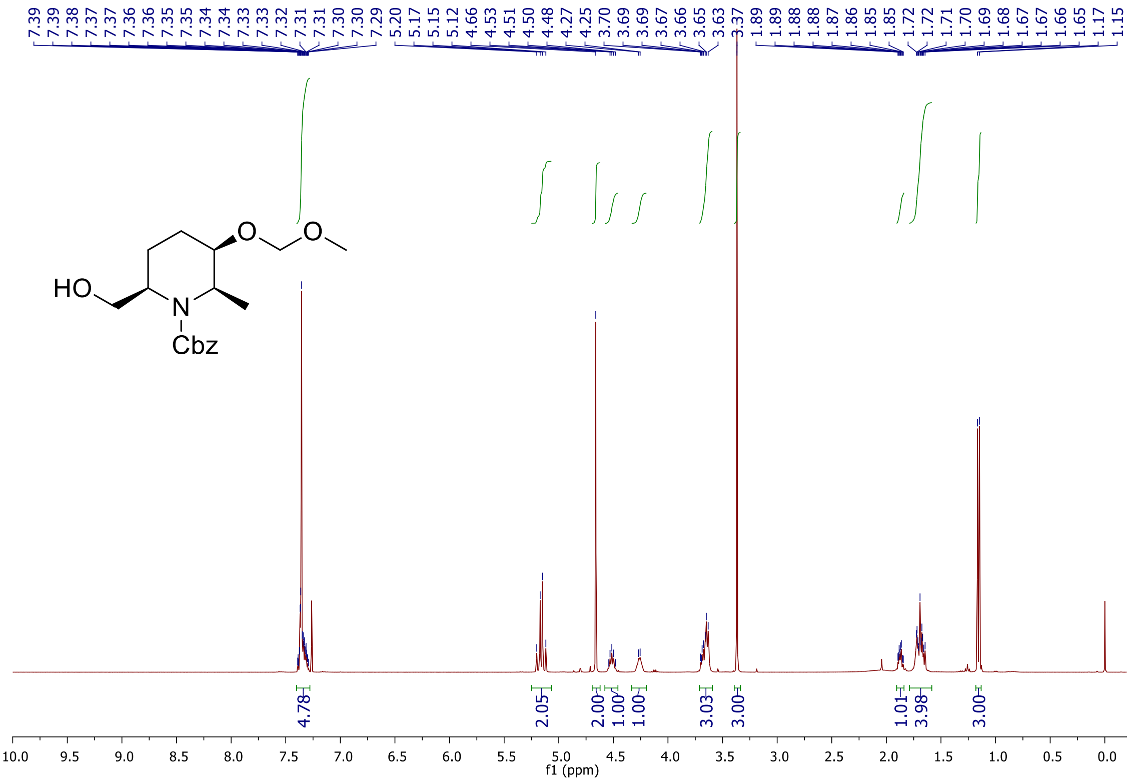

^{13}C{^{1}H} (101 MHz, CDCl$_3$, 26ºC) *(2R,3R,6R)-benzyl 6-(hydroxymethyl)-3-(methoxymethoxy)-2-methylpiperidine-1-carboxylate* **(19)**

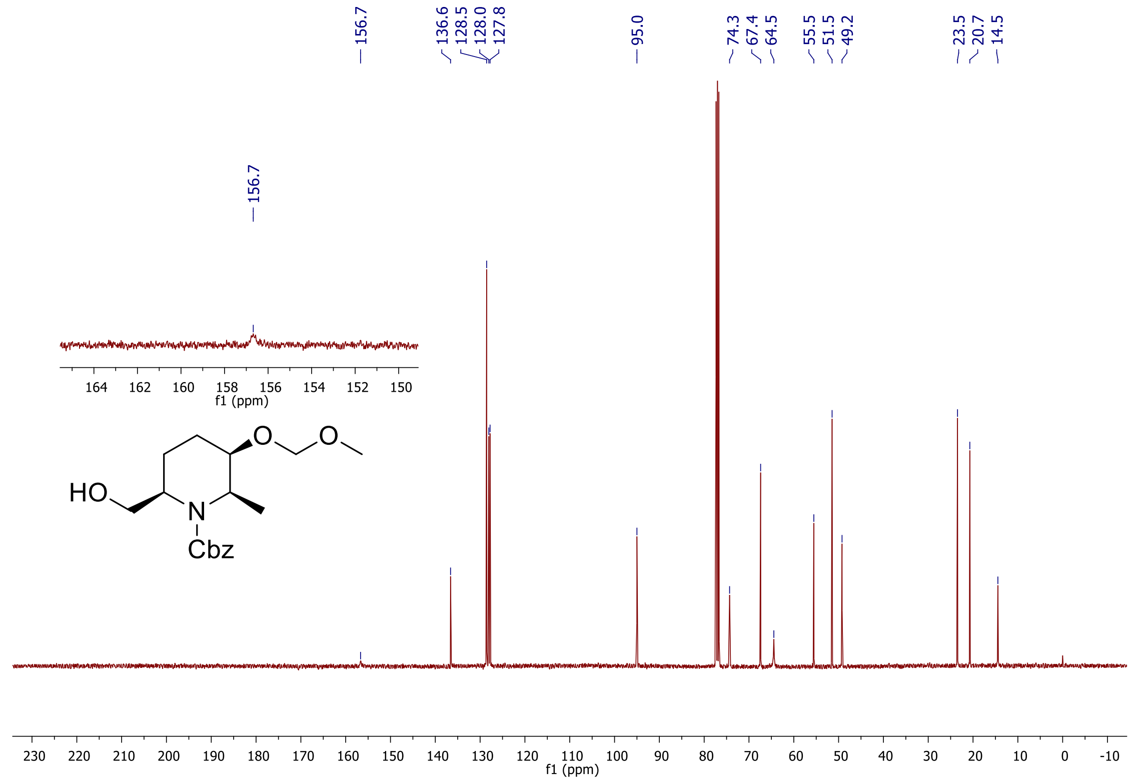

^{1}H (400 MHz, CDCl$_3$, 26°C) *(2R,3R,6R)-benzyl 6-((Z)-dodeca-2,11-dien-1-yl)-3-(methoxymethoxy)-2-methylpiperidine-1-carboxylate* **(22)**

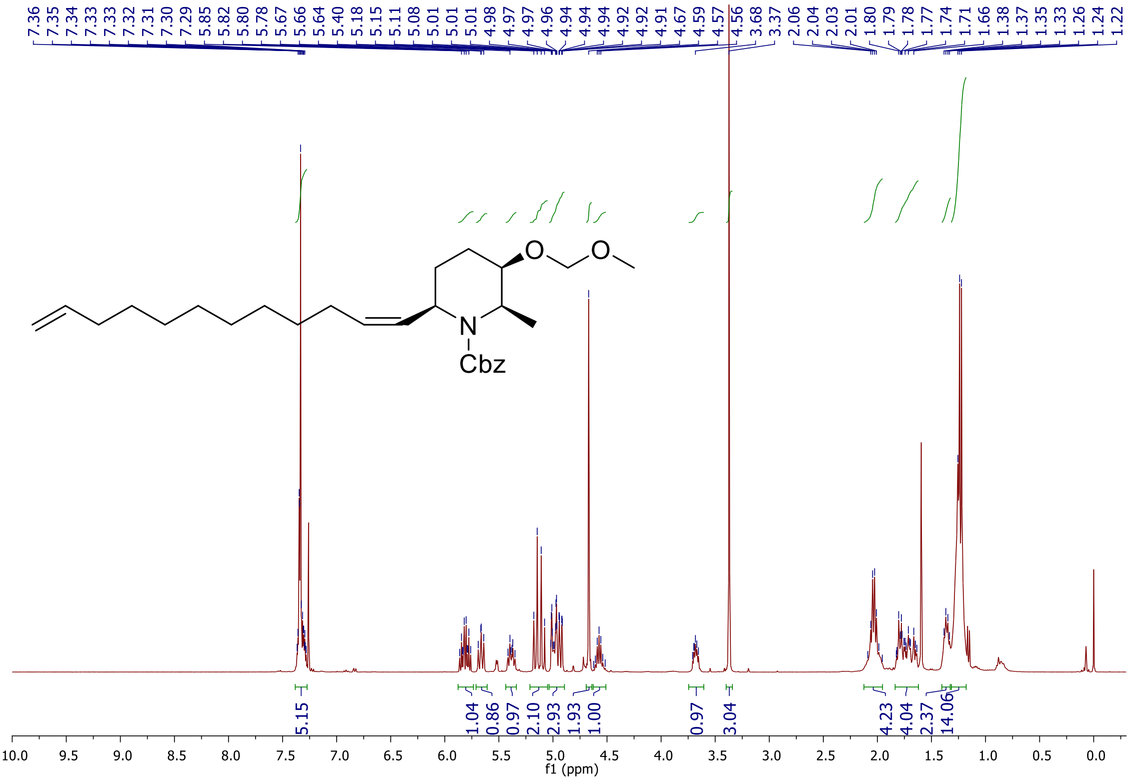

^{13}C{^{1}H} (101 MHz, CDCl$_3$, 26°C) *(2R,3R,6R)-benzyl 6-((Z)-dodeca-2,11-dien-1-yl)-3-(methoxymethoxy)-2-methylpiperidine-1-carboxylate* **(22)**

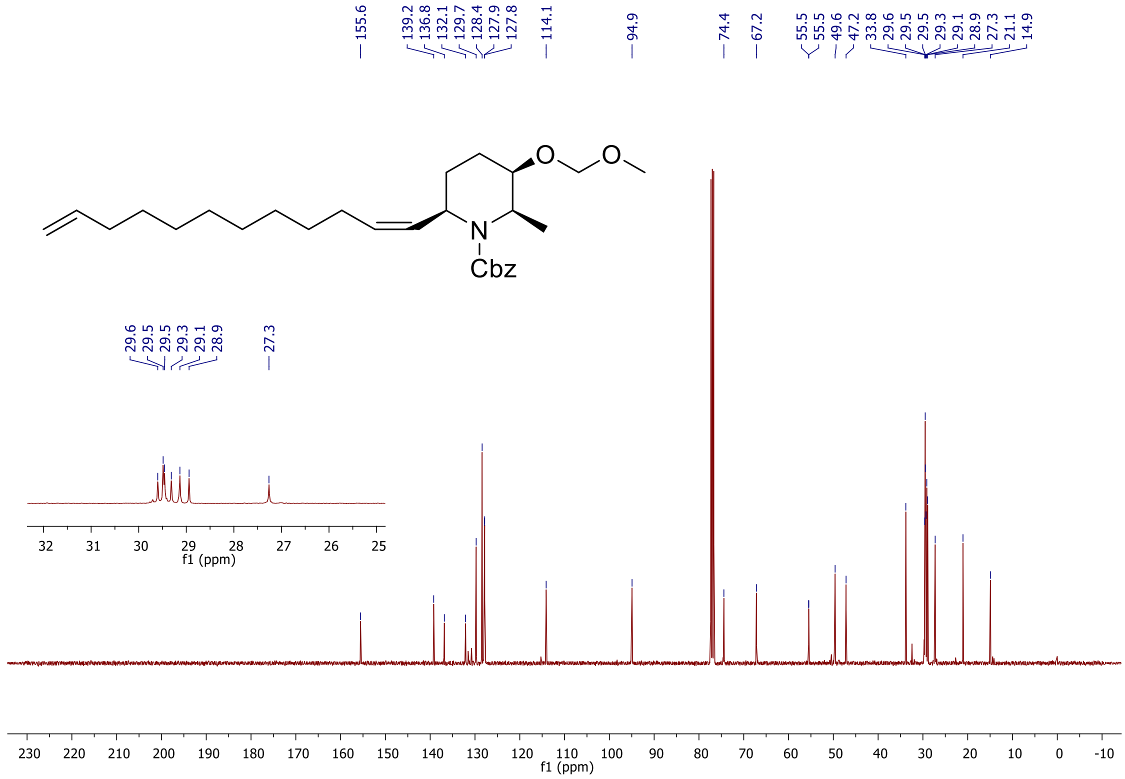

^{1}H (500 MHz, $CDCl_3$, 26ºC) *(2R,3R,6R)-benzyl 3-(methoxymethoxy)-2-methyl-6-((Z)-11-oxododec-2-en-1-yl)piperidine-1-carboxylate* **(23)**

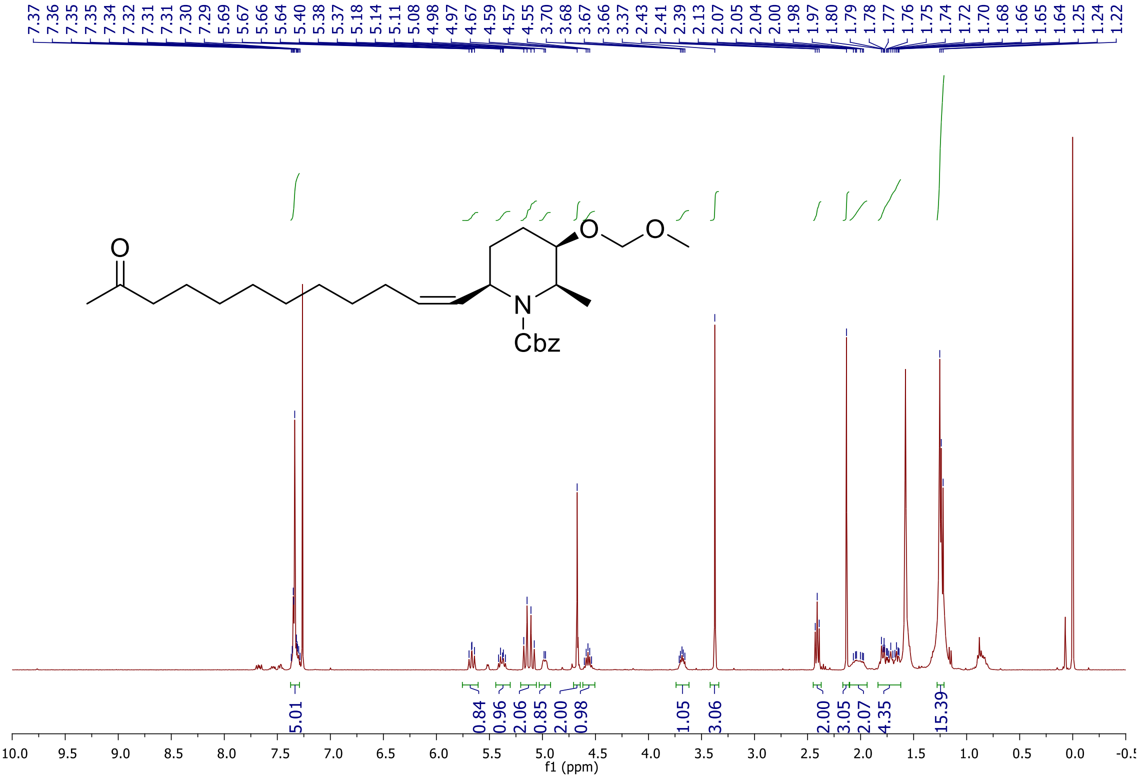

$^{13}C\{^{1}H\}$ (126 MHz, $CDCl_3$, 26ºC) *(2R,3R,6R)-benzyl 3-(methoxymethoxy)-2-methyl-6-((Z)-11-oxododec-2-en-1-yl)piperidine-1-carboxylate* **(23)**

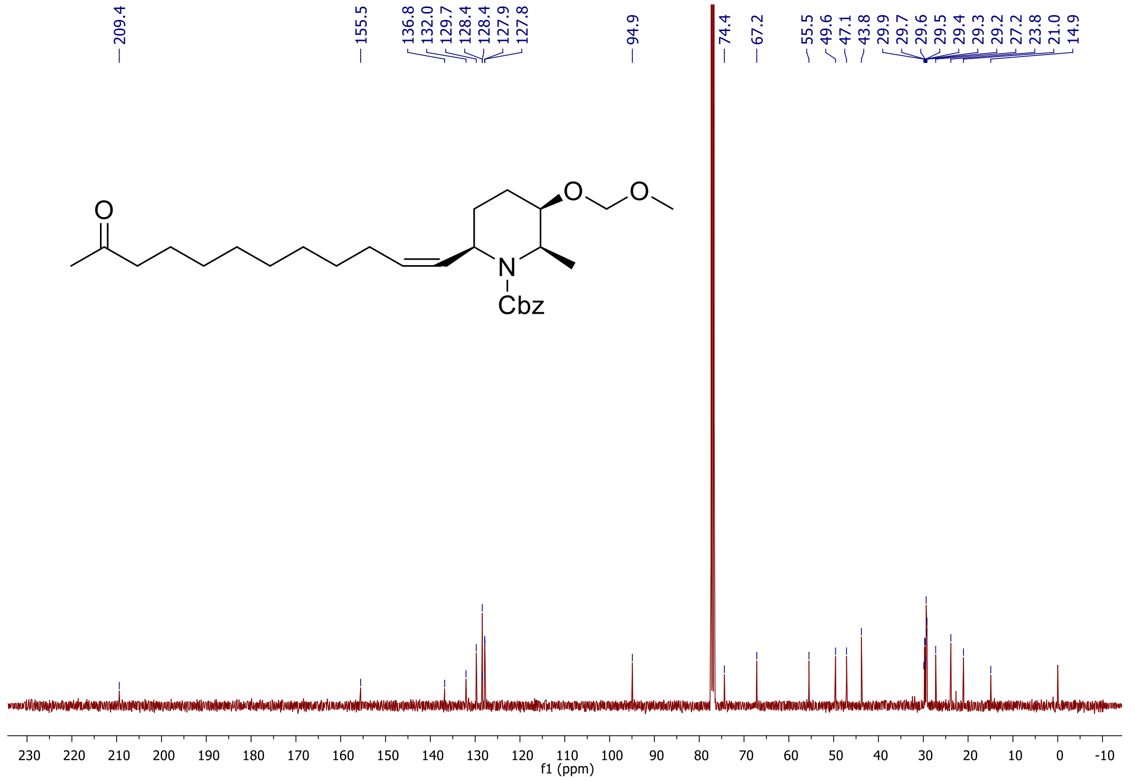

^{1}H (500 MHz, $CDCl_3$, 26ºC) (-)-Cassine (26)

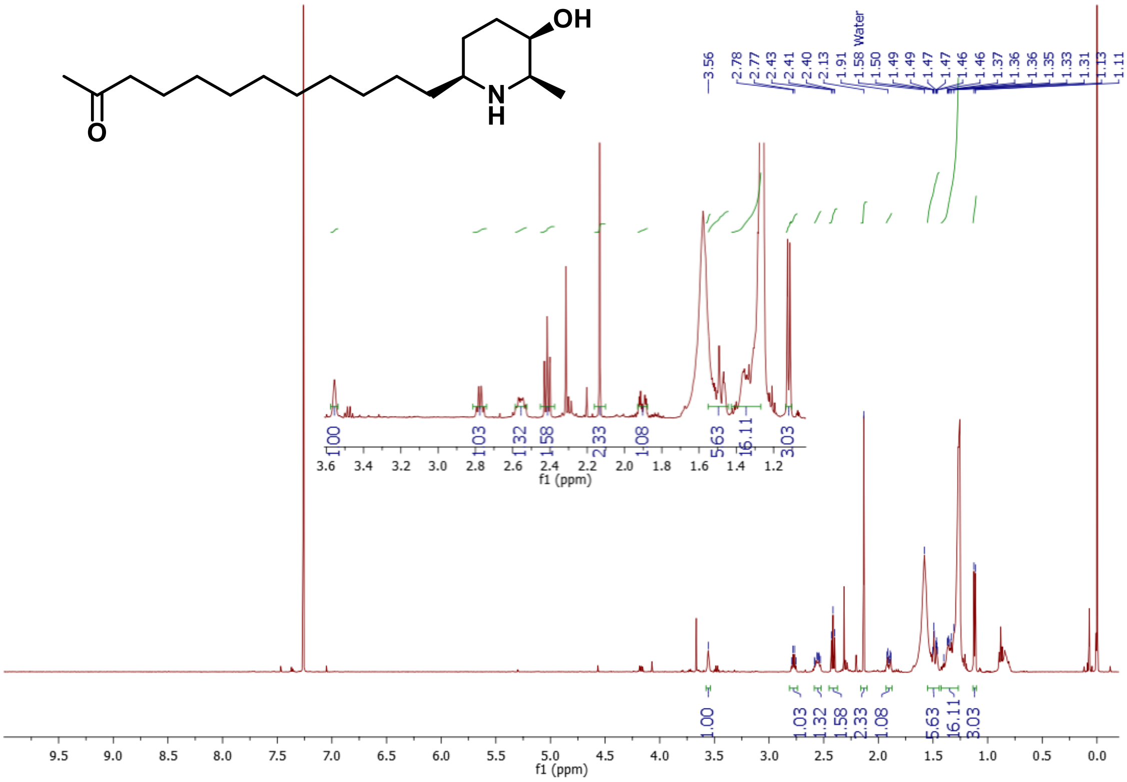

$^{13}C\{^{1}H\}$ (125 MHz, $CDCl_3$, 26ºC) (-)-Cassine (26)

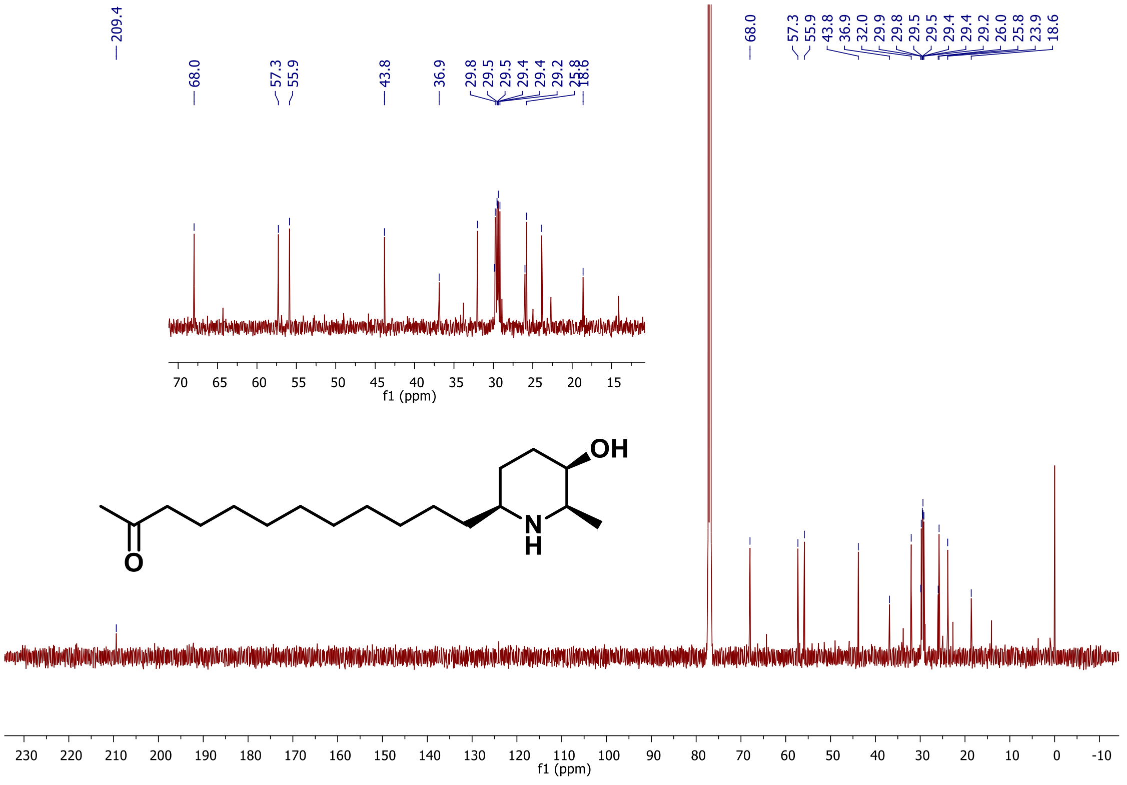

Table S3: ^{1}H NMR data vs literature.

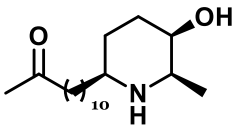

(-)-Cassine

^{1}H NMR (this work)	^{1}H NMR (literature)[Ref]
3.56 (broad singlet, 1H)	3.55 (broad singlet, 1H)
2.77 (broad quadruplet, *J* = 6.5 Hz, 1H)	2.76 (qd, *J* = 6.5/1.2 Hz, 1H)
2.55 (m, 1H)	2.54 (dddd, *J* = 11.4/5.8/5.62/5 Hz, 1H)
2.42 (t, *J* = 7.5 Hz, 2H)	2.42 (t, *J* = 7.5 Hz, 2H)
2.13 (s, 3H)	2.14 (s, 3H)
1.92-1.88 (m, 1H)	1.93-1.86 (m, 1H)
1.65-1.44 (m, 5H + water)	1.65-1.44 (m, 5H)
1.35-1.24 (m, 16H + “grease”)	1.35-1.24 (m, 16H)
1.11 (d, *J* = 6.5 Hz, 3H)	1.11 (d, *J* = 6.5 Hz, 3H)

Table S4: ^{13}C NMR data vs literature.

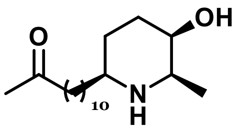

(-)-Cassine

^{13}C NMR (this work)	^{13}C{^{1}H} NMR (literature)^{Ref}
18.6	18.6
23.9	23.8
25.8	25.8
26.0	26.1
29.2	29.2
29.4	29.4
29.50 e 29.54	29.5 (2x)
29.8	29.7
29.9	29.8
32.0	32.0
36.9	37.0
43.8	43.8
55.9	55.8
57.3	57.2
68.0	68.0
209.4	209.4

NOESY1D Study of Compound (9)

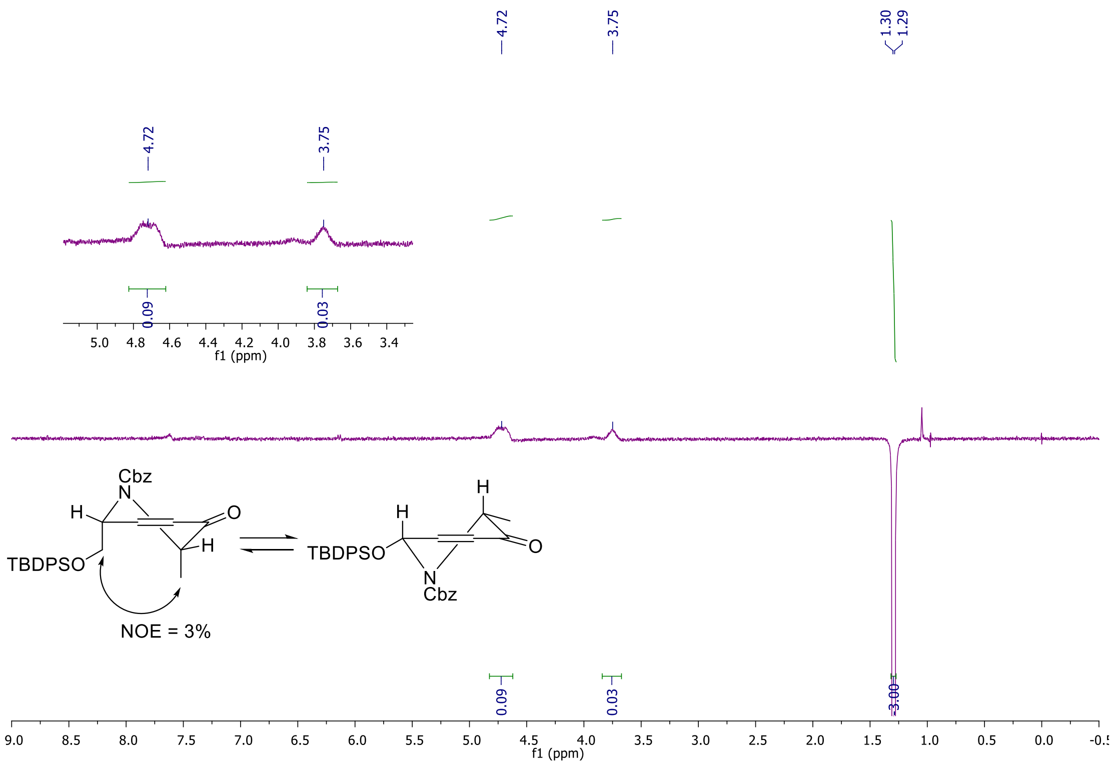

NOESY1D Study of Compound (12)

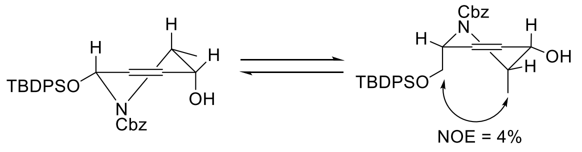

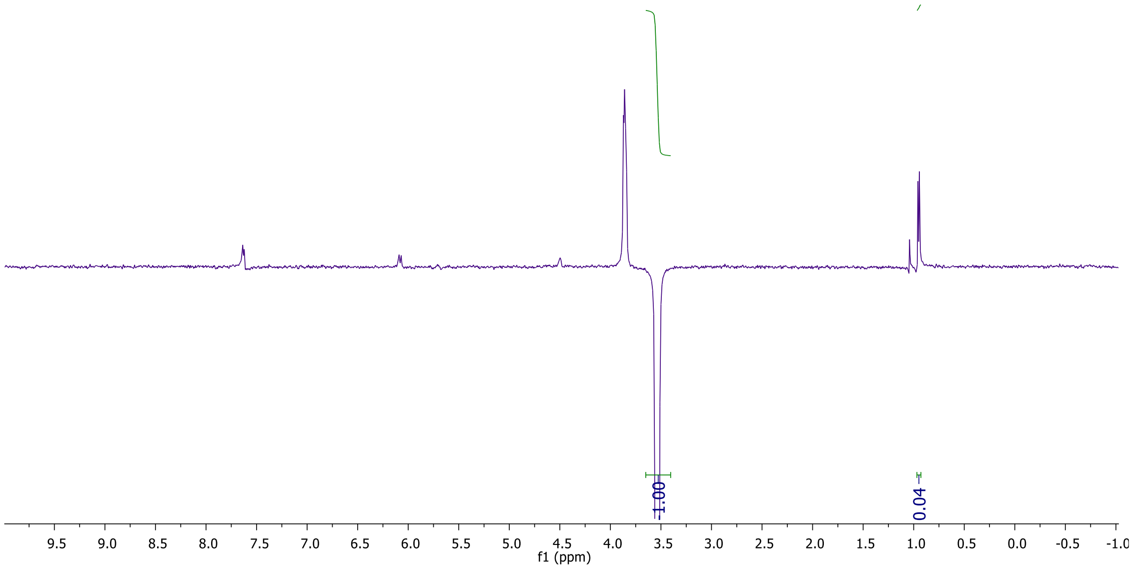

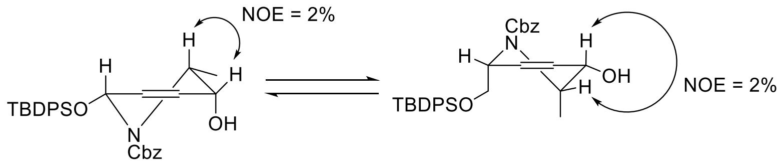

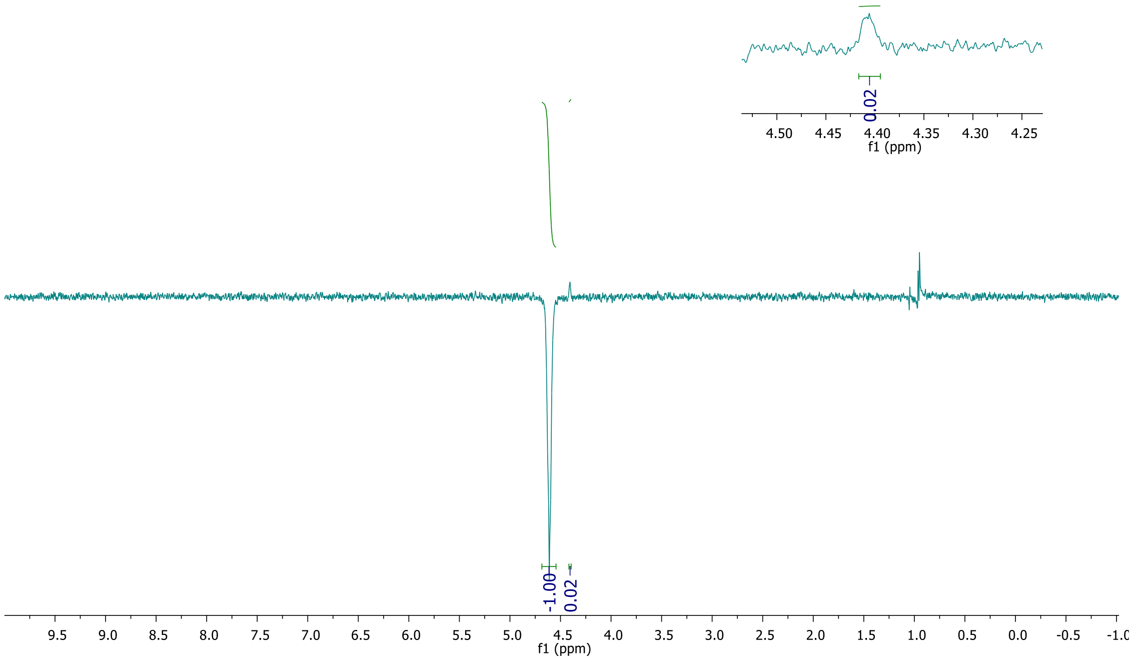

Theoretical calculations:

The geometries were initially optimized at the GFN2-xTB level of theory (xTB v6.4). Optimized geometries were subjected to a conformational search using CREST (v2.12) using the GFN2-xTB//GFN-FF composite method with CHCl3 solvation (ALPB model). These geometries were subjected to an additional optimization at GFN2-xTB level of theory with CHCl3 solvation (ALPB model). The geometries in the figure corresponds to those of lower energy. None of the ring conformation had a significant change in the 2 kcal/mol relative energy window.

P.Pracht, F.Bohle, S.Grimme, PCCP, 2020, 22, 7169-7192. b) S.Grimme, JCTC, 2019, 15, 2847-2862.

Cartesian Coordinates:

Structure 9:

E= -105.22502809, 0 imaginary frequency

C	1.3164394659	1.5214320372	0.2546982513
C	3.7074352381	1.7016379530	1.0412628099
C	2.8967917998	3.0560843786	-0.9140118660
C	1.6570644278	2.6286446027	-0.6734642215
H	0.8166839948	0.7395549905	-0.3317131167
H	3.1043822949	3.8247150488	-1.6434410150
H	0.8044388473	3.0612556923	-1.1753598740
H	4.5113381164	0.9834871629	1.2352051542
C	0.2763270533	1.9788357288	1.3115204956
H	0.2182945602	1.1953622484	2.0725929960
H	0.5961167511	2.9143161975	1.7803526537
O	-0.9791435349	2.2102013784	0.7584478081
Si	-2.0177477008	1.1886023326	-0.0097665383
C	-3.5906816382	2.2894827124	-0.1440179683
C	-3.2694758010	3.7062130950	-0.6236923999

H	-4.1683523557	4.3229648110	-0.6029179708
H	-2.5233866970	4.1645735838	0.0196832745
H	-2.8912883813	3.7035406644	-1.6419885293
C	-4.2480596421	2.3965988473	1.2361651702
H	-5.1480764002	3.0090775787	1.1800815720
H	-4.5283171160	1.4150577876	1.6120271473
H	-3.5673771163	2.8565184357	1.9483590437
C	-4.5876213844	1.6437564567	-1.1066300037
H	-4.8729937438	0.6554320317	-0.7538987353
H	-5.4903680109	2.2497332271	-1.1840259097
H	-4.1605330664	1.5391896950	-2.1009090821
C	-2.4235830592	-0.3524744454	1.0233513634
C	-2.9906013032	-1.4876305594	0.4464115239
C	-2.1936021355	-0.3727410990	2.3970133259
C	-3.3016465742	-2.6017319505	1.2063064277
H	-3.1889757005	-1.4967989151	-0.6152429821
C	-2.4936686579	-1.4857347032	3.1634240293
H	-1.7837327359	0.5032833385	2.8777406372
C	-3.0473728208	-2.6073481336	2.5684848305
H	-3.7443511544	-3.4692335015	0.7368511465
H	-2.2997205513	-1.4783924740	4.2269960681
H	-3.2826947731	-3.4783639120	3.1632009170
C	-1.2804206448	0.5802282333	-1.6604103670
C	-0.6442483257	-0.6600018309	-1.7272457075
C	-1.2951183816	1.3554233718	-2.8173552500
C	-0.0523569957	-1.1074926327	-2.8949013099
H	-0.6055151685	-1.2860155356	-0.8486459370

C	-0.7068001114	0.9154838053	-3.9905920845
H	-1.7732502404	2.3225233888	-2.8030868832
C	-0.0826986180	-0.3198348023	-4.0337138693
H	0.4333387495	-2.0725497462	-2.9111262323
H	-0.7345575728	1.5377374161	-4.8738277405
H	0.3772669027	-0.6662761661	-4.9477656530
C	4.0458984828	2.5059647026	-0.2049057463
O	5.2000029167	2.7321329979	-0.5188767325
C	3.6213201513	2.6769584319	2.2239703986
H	4.5849283597	3.1608928795	2.3600724020
H	2.8679655408	3.4380529054	2.0401719806
H	3.3712076560	2.1289609353	3.1286184072
N	2.4860186314	0.9313143170	0.8741434622
C	2.4577381124	-0.3376736703	1.3358446900
O	3.4055945872	-0.8988317023	1.8540346732
O	1.2471875923	-0.9085320439	1.1695600379
C	1.0966028737	-2.2799160035	1.5759381356
H	1.8933436272	-2.5188088874	2.2886263684
H	0.1211655056	-2.3300963233	2.0648258683
C	1.1517864402	-3.1990982897	0.3849766007
C	0.0039943709	-3.8352536519	-0.0694863786
C	2.3524633744	-3.3962178220	-0.2870983625
C	0.0571566820	-4.6626732053	-1.1782762117
H	-0.9309125331	-3.6804544430	0.4486555201
C	2.4033697355	-4.2196942049	-1.3982447848
H	3.2465267712	-2.9028325875	0.0672488908
C	1.2559430927	-4.8552143146	-1.8446602225

H	-0.8397073793	-5.1544175095	-1.5246054599
H	3.3410022424	-4.3666778179	-1.9137652120
H	1.2972829934	-5.4988638899	-2.7110709385

Structure 12:

E = -106.25335548, 0 imaginary frequency

C	-1.4332545842	1.3944684316	-0.2746657857
C	-3.8331920537	1.3475888679	-1.0314598142
C	-3.1584795401	2.7272865370	0.9158393892
C	-1.8916751780	2.5094149133	0.6083884773
H	-0.8862921516	0.6720442756	0.3432792543
H	-3.4620623862	3.5336212911	1.5660720901
H	-1.0936379724	3.1244704983	0.9931555974
H	-4.5369349637	0.5771247057	-1.3619357281
C	-0.4280641321	1.9038763790	-1.3328326119
H	-0.2880412510	1.1106552326	-2.0728079752
H	-0.8199189398	2.7955269807	-1.8317752465
O	0.7992008806	2.2607892703	-0.7744497954
Si	1.9191115974	1.3480100375	0.0100783551
C	3.3849491286	2.5894544356	0.1410007451
C	4.4356301402	2.0425255800	1.1074316443
H	4.0182719515	1.9029857490	2.1014676765
H	4.8106885871	1.0830479508	0.7590331281
H	5.2788577725	2.7292164335	1.1832533445
C	2.9337136660	3.9725757075	0.6134671895
H	2.1536612671	4.3585298262	-0.0371216180
H	2.5483821092	3.9380785805	1.6285186664

H	3.7728080286	4.6687655723	0.5970498631
C	4.0325866075	2.7509359861	-1.2383829509
H	4.8709490137	3.4457083368	-1.1842935016
H	4.4043170628	1.7978901015	-1.6081565211
H	3.3128903188	3.1405865909	-1.9541321725
C	2.4785739716	-0.1548778975	-1.0091482435
C	2.2669316557	-0.2026935301	-2.3849564896
C	3.1408044545	-1.2306087853	-0.4201918137
C	2.6766393595	-1.2866809693	-3.1421833911
H	1.7823738748	0.6295227262	-2.8741481662
C	3.5615967447	-2.3148026424	-1.1705187912
H	3.3264361109	-1.2168899098	0.6437672101
C	3.3245756451	-2.3498580544	-2.5353211470
H	2.4943954020	-1.3019268579	-4.2078136547
H	4.0764063804	-3.1360213073	-0.6913489969
H	3.6455866137	-3.1982168157	-3.1227303677
C	1.2462936129	0.6782403815	1.6639719988
C	1.2029332987	1.4476029551	2.8236346455
C	0.7145136141	-0.6100443909	1.7290167349
C	0.6576132365	0.9570306022	3.9975691524
H	1.5988293392	2.4511431862	2.8107064241
C	0.1665759301	-1.1088083725	2.8971191758
H	0.7214812070	-1.2327442889	0.8471181969
C	0.1372245291	-0.3252876041	4.0388303116
H	0.6376013450	1.5770214976	4.8826658266
H	-0.2386115930	-2.1104756618	2.9112129790
H	-0.2893300847	-0.7112343064	4.9533393913

C	-3.7948103271	2.4656913727	-2.0736267305
H	-3.3589605646	2.0863243645	-2.9942143498
H	-4.8069784475	2.7977366404	-2.2908850500
H	-3.2043081014	3.3074404292	-1.7207586319
N	-2.5409908301	0.6917567323	-0.8999602348
C	-2.3936168651	-0.5679569581	-1.3534269228
O	-3.2816649661	-1.2201549372	-1.8742033333
O	-1.1347797823	-1.0265487825	-1.1820122030
C	-0.8590913745	-2.3775336597	-1.5885974484
H	-1.6270401879	-2.6856395812	-2.3065715889
H	0.1199811610	-2.3395884554	-2.0716180489
C	-0.8391124212	-3.3001690087	-0.3991331127
C	0.3583885479	-3.8286441637	0.0648202176
C	-2.0221359340	-3.6079749914	0.2625582257
C	0.3722408544	-4.6588289021	1.1727548673
H	1.2794605039	-3.5872273301	-0.4450071381
C	-2.0064737595	-4.4341445682	1.3727277878
H	-2.9541599407	-3.1977782711	-0.0998800859
C	-0.8092828460	-4.9619267940	1.8286760485
H	1.3077939303	-5.0659671714	1.5265243104
H	-2.9308700258	-4.6677257681	1.8801679388
H	-0.7983601800	-5.6076079330	2.6945233447
C	-4.2571043240	1.8630789185	0.3646547108
H	-4.4086659116	0.9974598838	1.0234431756
O	-5.5057916241	2.5156744138	0.3416705889
H	-5.4100092572	3.3325731680	-0.1665785244
